# Doxorubicin induces cardiotoxicity in a pluripotent stem cell model of aggressive B cell lymphoma cancer patients

**DOI:** 10.1007/s00395-022-00918-7

**Published:** 2022-03-08

**Authors:** Luis Peter Haupt, Sabine Rebs, Wiebke Maurer, Daniela Hübscher, Malte Tiburcy, Steffen Pabel, Andreas Maus, Steffen Köhne, Rewati Tappu, Jan Haas, Yun Li, Andre Sasse, Celio C. X. Santos, Ralf Dressel, Leszek Wojnowski, Gertrude Bunt, Wiebke Möbius, Ajay M. Shah, Benjamin Meder, Bernd Wollnik, Samuel Sossalla, Gerd Hasenfuss, Katrin Streckfuss-Bömeke

**Affiliations:** 1 Clinic for Cardiology and Pneumology, University Medical Centre Göttingen, Göttingen, Germany; 2 DZHK (German Centre for Cardiovascular Research), partner site Göttingen, Göttingen, Germany; 3 King’s College London, British Heart Foundation Centre of Excellence, London, UK; 4 Institute of Pharmacology and Toxicology, University Medical Centre Göttingen, Göttingen, Germany; 5 Department of Internal Medicine 2, Cardiology, University Medical Centre Regensburg, Regensburg, Germany; 6 Department of Cardiology, University of Heidelberg, Heidelberg, Germany; 7 Institute of Human Genetics, University Hospital Centre Göttingen, Göttingen, Germany; 8 Institute of Cellular and Molecular Immunology, University Medical Centre Göttingen, Göttingen, Germany; 9 Department of Pharmacology, University Medical Centre Mainz, Mainz, Germany; 10 Clinical Optical Microscopy, University Medical Centre Göttingen, Göttingen, Germany; 11 Department of Neurogenetics, Electron Microscopy Core Unit, Max Planck Institute for Multidisciplinary Sciences, Göttingen, Germany; 12 Cluster of Excellence “Multiscale Bioimaging: From Molecular Machines to Networks of Excitable Cells” (MBExC), University of Göttingen, Göttingen, Germany; 13 Institute of Pharmacology and Toxicology, Würzburg University, Würzburg, Germany; 14 DZHK (German Centrefor Cardiovascular Research), partner site Heidelberg, Heidelberg, Germany

**Keywords:** Anthracyclin-induced cardiotoxicity (ACT), Induced pluripotent stem cells (iPSC), Cardiomyocytes, Cardiac fibroblasts, Heart failure, Doxorubicin

## Abstract

Cancer therapies with anthracyclines have been shown to induce cardiovascular complications. The aims of this study were to establish an in vitro induced pluripotent stem cell model (iPSC) of anthracycline-induced cardiotoxicity (ACT) from patients with an aggressive form of B-cell lymphoma and to examine whether doxorubicin (DOX)-treated ACT-iPSC cardiomyocytes (CM) can recapitulate the clinical features exhibited by patients, and thus help uncover a DOX-dependent pathomechanism. ACT-iPSC CM generated from individuals with CD20^+^ B-cell lymphoma who had received high doses of DOX and suffered cardiac dysfunction were studied and compared to control-iPSC CM from cancer survivors without cardiac symptoms. In cellular studies, ACT-iPSC CM were persistently more susceptible to DOX toxicity including augmented disorganized myofilament structure, changed mitochondrial shape, and increased apoptotic events. Consistently, ACT-iPSC CM and cardiac fibroblasts isolated from fibrotic human ACT myocardium exhibited higher DOX-dependent reactive oxygen species. In functional studies, Ca^2+^ transient amplitude of ACT-iPSC CM was reduced compared to control cells, and diastolic sarcoplasmic reticulum Ca^2+^ leak was DOX-dependently increased. This could be explained by overactive CaMKIIδ in ACT CM. Together with DOX-dependent augmented proarrhythmic cellular triggers and prolonged action potentials in ACT CM, this suggests a cellular link to arrhythmogenic events and contractile dysfunction especially found in ACT engineered human myocardium. CamKIIδ inhibition prevented proarrhythmic triggers in ACT. In contrast, control CM upregulated SERCA2a expression in a DOX-dependent manner, possibly to avoid heart failure conditions. In conclusion, we developed the first human patient-specific stem cell model of DOX-induced cardiac dysfunction from patients with B-cell lymphoma. Our results suggest that DOX-induced stress resulted in arrhythmogenic events associated with contractile dysfunction and finally in heart failure after persistent stress activation in ACT patients.

**Supplementary Information:**

The online version contains supplementary material available at 10.1007/s00395-022-00918-7.

## Introduction

Doxorubicin (DOX) has an outstanding efficacy against a broad range of solid and hematopoietic cancers. However, its clinical use is restricted to its high risk for anthracycline-induced cardiotoxicity (ACT). The lifelong cumulative dose of DOX was set at 500 mg/m^2^, but the incidence of ACT is still at 5–9%, with up to 18% of DOX-treated patients showing subclinical symptoms [5, 37]. The side effects of cardiotoxicity include disturbance in ventricular de-/ repolarization, arrhythmia, decrease in left ventricular ejection fraction (LVEF), and fractional shortening (FS) often leading to dilative heart failure and enhanced mortality [25]. Although ACT causes lethal congestive heart failure, no early detection or effective treatment methods are yet available [42]. Development of ACT is highly inter-individually variable and is thus not possible to accurately predict its occurrence. The reason, why only some patients develop ACT, and exactly why it developed in these and not others, has been the subject of intense study. Recently, clinical study cohorts were investigated for an association of gene variants with ACT [33, 48] including the ‘rituximab with CHOP over age 60 years’ (RICOVER60) trial [29] and the NHL-B1/B2 study.

The pathophysiology of ACT is described as multifactorial, and the exact causal molecular mechanisms of DOX-induced cardiotoxicity remain elusive. Generation of ROS by so-called redox cycling in cardiomyocytes can cause lipid peroxidation, DNA damage, and mitochondrial dysregulation. Mitochondria are the major site of DOX-dependent ROS production due to the localization of redox cycling enzymes such as NADH oxidases [18]. Dysregulated redox signaling is known to be connected to cardiac diseases [3]. Other proposed mechanisms include inhibition of topoisomerase II by DOX’ intercalation into DNA, leading to DNA damage-induced cellular stress and cell death in cardiomyocytes [8], which in turn results in loss of functional cardiomyocytes and heart injury [50]. In addition, DOX can influence the epigenetic status of the DNA either directly by altering the expression of epigenetic enzymes involved in DNA methylation, or histone modification or indirectly due to its damage to mitochondrial function and therefore disruption of mitochondrial metabolite production [23, 44]. Finally, DOX induces disruption of Ca^2+^ homeostasis in CM by increasing Ca^2+^ release from the sarcoplasmic reticulum (SR) by higher opening probabilities of ryanodine receptor 2 (RYR2) [16, 35]. On the other hand, expression and activity of the sarco-/ endoplasmic reticulum Ca^2+^ATPase (SERCA2a) is reduced by DOX [16, 52], leading to decreased Ca^2+^ transport into the SR and cytoplasmic Ca^2+^ overload. This results in sarcomeric disarray and a reduction in the heart’s contractile force and may contribute to the generation of arrhythmia [16, 17].

Not only cardiomyocytes but also cardiac fibroblasts (cFB) are key players in myocardial pathology. cFB are considered the predominant source of the extracellular matrix and the hallmark of fibrosis [45]. During cardiac stress, cFB trans-differentiate into myofibroblasts, which perform cardiac extracellular remodeling and gain contractile activity due to alpha-smooth muscle (α-SMA) expression [49]. An increase in the extracellular matrix (ECM) normally results in cardiac stiffness and diastolic dysfunction as well as impaired cardiac contraction [7]**.** Still, less is known about the direct effects of DOX on human cFB from diseased ACT myocardium.

There has been a lack of human cardiomyocyte culture models from patients with ACT that could be used to better understand the molecular and cellular physiology of ACT. However, the generation of induced pluripotent stem cell-derived cardiomyocytes (iPSC CM) has now made it possible to establish ACT patient-specific disease models. iPSC CM have been widely used to study hereditary cardiac conditions and demonstrated close imitation of clinical drug responses in vitro, including arrhythmic disorders or cardiomyopathies, with a correlation to predicted phenotypes [28, 31, 36]. Patient-specific stem cell models showed a predilection of breast cancer patients to low DOX-induced cardiotoxicity [4] or trastuzumab-induced cardiac dysfunction [21]. General cardiotoxic effects of anthracyclines in cardio-oncology have been also shown in healthy control-iPSC CM including apoptosis, ROS production and mitochondrial dysfunction, as well as electrophysiological and Ca^2+^ handling aberrations [20, 27]. A genetic predisposition has been suggested for ACT [48], including multiple somatic and systemic mutations triggering the development of ACT [32]. Recently, a stem cell model of pediatric ACT patients identified drug transporter genomic variants associated with DOX-induced cardiotoxicity [26].

In the present study, we developed the first human iPSC CM model of DOX-induced cardiac dysfunction in patients with aggressive B cell lymphoma and showed that ACT-cells are persistently more susceptible to detrimental effects of DOX treatment compared to controls. On a patient-specific level, we demonstrated that control cells upregulated SERCA2a expression in a DOX-dependent manner to avoid cytoplasmic Ca^2+^ overload and heart failure conditions, whereas the hyperactivation of Ca^2+^/calmodulin-dependent protein kinase II δ (CaMKIIδ) was unique for ACT patients, resulting in arrhythmogenic triggers and contractile dysfunction.

## Material and methods

More details are available in the Supplementary Appendix.

### Human myocardium of ACT patients

We obtained left ventricular tissues from explanted hearts of 5 ACT patients with end-stage HF who were undergoing heart transplantation (EF ≤ 20%). After explantation, the heart was immediately placed in pre-cooled cardioplegic solution containing NaCl (110 mM), KCl (16 mM), MgCl2 (16 mM), NaHCO3 (16 mM), CaCl2 (1.2 mM), and glucose (11 mM). Myocardial samples for qPCR, Western blot and immunocytochemistry stainings (fibrosis) were immediately frozen in liquid nitrogen and stored at − 80 °C. We used control myocardium from healthy donors’ hearts that could not be transplanted for technical reasons for qPCR, immunoblots and fibrotic stainings. Collection of human samples was in compliance with the local ethical committee (Az. 31/9/00) and written informed consent was received from each participant before transplantation.

### Selection of ACT patients for iPSC generation

Patients described in this study were initially identified as lymphoma patients and selected from the ‘rituximab with CHOP over age 60 years’ (RICOVER60) trial (NCT00052936). The study cohorts comprised 61–80-year-old patients with aggressive CD20 + B cell lymphomas treated with six or eight cycles of CHOP-14 with or without rituximab. All patients of this study had been treated with doxorubicin (DOX) as part of a CHOP treatment. ACT patients 1–3 suffered from anthracycline-induced cardiotoxicity, whereas control patients did not develop cardiac symptoms.

### Human skin and cardiac fibroblast isolation and cultivation

The study was approved by the ethical committee of the University Medical Center Göttingen (Az 21/1/11 and 31/9/00), and written informed consent was received from each participant. The fibroblast culture was derived from skin punch biopsies of the donors (RICOVER60 study) or from human cardiac tissue (approximately 1 mm^3^) from the left ventricle of ACT end-stage heart failure patients. Somatic cells were isolated as previously described [2]. To secure attachment and proliferation of the fibroblasts, the dish was left without medium change for one week, after which the tissue was removed. Primary skin or cardiac fibroblasts were cultured in fibroblast growth medium composed of DMEM supplemented with 10% FCS, 1 × NEAA, glutamine (2 mmol/L), β-ME (50 µmol/L), penicillin (50 U/mL)/streptomycin (50 µg/mL), and bFGF (10 ng/mL) at 37 °C with 5% CO_2_ atmosphere. The medium was replaced every second day and cells were passaged once to twice per week. FB were used from passage 3–6.

### Quantification of fibrosis in human cardiac tissue

The frozen cardiac tissue of ACT patients, as well as healthy controls, was embedded, sliced, and stained with Masson’s trichrome staining in cooperation with the Department of Pathology of the University Medical Center Göttingen. The area of fibrosis was quantified in cooperation with the Technology Platform Clinical Optical Microscopy. The slides were first scanned for virtual microscopy using a 20 × objective (UPlanApo) with the dotSlide SL slide scanner (Olympus) and a peltier-cooled XC10 camera. Fibrotic areas were then calculated by a scientist blinded to analyzed myocardium, using the CellSens Dimensions software (Olympus).

### Human-induced pluripotent stem cell derivation and culture

Human dermal fibroblasts of five lymphoma patients were reprogrammed into iPSC (Supplementary Table 6) as described earlier [2]. Briefly, 3 × 10^5^ fibroblast were seeded in 2 wells of a 6-well or 12-well plate one day before transduction. Sendai virus from the CytoTune™-iPS 2.0 Sendai Reprogramming Kit (Thermo Fisher Scientific) was used at a MOI of 5/5/3 (KOS/hc-myc/hKLF4) and added to the cells in a fresh medium. At day 7 post transduction, fibroblasts were harvested and plated on a Geltrex-coated 6 well plates in E8 medium (Thermo Fisher Scientific) for picking iPSC-like colonies 2–3 weeks later. All cell lines were free of mycoplasma contamination, which were performed every 10 passages via luminescence with the Lonza Mycoalert Plus-Kit.

### Directed cardiac differentiation and cardiomyocyte culture

For each donor, iPSC of two cell lines were directly differentiated in vitro into pure iPSC CM based on published protocols [2]. This was achieved by sequential targeting of the WNT pathway with the GSK3 inhibitor CHIR99021 (4 µmol/L, Millipore) in RPMI 1640 medium (Thermo Fisher Scientific) supplemented with 0.02% L-Ascorbic Acid 2-Phosphate (Sigma Aldrich) and 0.05% albumin (Sigma Aldrich) for 48 h, followed by incubation with 5 µmol/L of the inhibitor of Wnt production-2 (IWP2, Millipore) for another 48 h. When the first contractions were visible, the medium was changed to RPMI 1640 medium supplemented with 2 mmol/L L-glutamine and B27 with insulin (Life Technologies). Three to four weeks after the start of the differentiation the cells were metabolically selected by replacing L-glutamine with 4 mmol/L lactate as a carbon source. If not otherwise indicated, 2-month-old iPSC CM were exposed for 24 h to DOX and analyzed regarding the different readouts.

### Intracellular DOX accumulation

Intracellular DOX levels were analyzed according to an adapted protocol for intracellular DHE measurement [12]. In short, DOX-challenged iPSC CM were washed twice with PBS + 100 μM diethylenetriaminepentaacetic acid (DTPA; Sigma-Adlrich) and immediately lysed with 100% HPLC-grade acetonitrile (Milipore) using 500 µl per well. Lysates were centrifuged at 10,000×*g* for 10 min in a cooled table top centrifuge (4 °C, Eppendorf 5418 R). The supernatant was transferred to a fresh tube, dried in a vacuum concentrator (Thermo Fisher Scientific), resuspended in PBS + DTPA and measured with HPLC (Dionex). The injection volume was 25 µl per sample and the flow rate was set to 0.400 ml/min with a pressure of approximately 70 bar. Solution A was > 99% acetonitrile with 0.1% trifluoroacetic acid (TFA; Sigma-Aldrich) and solution B was Milipore grade I-filtered water with 0.1% TFA. Initial solution A to solution B ratio was 85%:15%, gradually shifting to 15%:85% over the measurement period of 40 min. DOX peaks were identified by a reference injection of diluted DOX and appeared after a retention time of approximately 16 min. For each peak, the area under the curve was considered. Peak analysis was carried out with Chromeleon 7 software.

### Ca^2+^ imaging

Ca^2+^ was measured as described before [2]. In short, 2.5 × 10^5^ cells were plated onto a round 25 mm glass cover-slip and cultured for one week. On the day of imaging, the cells were incubated with 5 µM Fluo-4 in tyrode solution for 30 min in the dark. After washing the cells with tyrode solution, images of the cells were captured with a Carl Zeiss LSM 710 confocal microscope and a 63 × 1.4 NA oil objective in line scan mode (512 pixels, 45.5 µm, 1057.7 Hz, 20,000 cycles). The fluorescence intensities were processed with FIJI software, Prism 6 (GraphPad), and LabChart (ADInstruments). For analysis of the calcium sparks, the FIJI plugin “Sparkmaster” was used with the following criteria: scanning speed: 1057.7, pixelsize: 0.088, background FI.U.: 0, criteria: 3.3 and no. of intervals: 3. The spark analysis was performed in the last diastole (rectangle height 455, width 1058) of each line scan. To calculate the calcium leakage, the product of amplitude, width and height for each spark was determined.

### Action potential recordings

Whole-cell patch-clamp technique (current-clamp configuration) was used for action potential recordings of iPSC CM. Microelectrodes (3–5 MΩ) were filled with (in mmol/L): 92 K-Aspartate, 48 KCl, 1 Mg-ATP, 10 HEPES, 0.02 EGTA, 0.1 GTP-Tris, and 4 Na2-ATP (pH 7.2, KOH). The bath solution contained (in mmol/L): 140 NaCl, 4 KCl, 1 MgCl2, 1.25 CaCl2, 10 Glucose, and 10 HEPES (pH 7.4, NaOH). After rupture, access resistance was typically 5–15 MΩ. Action potentials of iPSC-CM were continuously elicited by square current pulses of 1 nA amplitude and 2–5 ms duration at a frequency of 0.5 Hz. Fast capacitance was compensated in cell-attached configuration. Membrane capacitance and series resistance were compensated after patch rupture. Signals were filtered with 2.9 and 10 kHz Bessel filters and recorded with an EPC10 amplifier (HEKA Elektronik). Measurements were performed at room temperature.

### Analysis of delayed afterdepolarizations

For investigation of the incidence of delayed afterdepolarizations (DADs) in iPSC-CM, membrane potential was measured using the whole-cell patch-clamp technique (current-clamp configuration). DADs were analyzed during rest intervals after stimulated action potentials (1 Hz, Fig. 7E, F) or during prolonged rest intervals (10 s pause, Fig. 7C, D). Depolarizations with an amplitude > 5 mV were counted manually and the DAD incidence per minute was calculated.

### 3D-culture of iPSC CM

Engineered human myocardium (EHM) was generated as described previously [38]. In short, cardiomyocytes and human foreskin fibroblasts were mixed at a ratio of 70/30% as previously described [38] and cultured for 3 days in Iscove-Medium with 4% B27 without insulin, 1% non-essential amino acids, 2 mmol/l glutamine, 300 µmol/l ascorbic acid, 100 ng/ml IGF1 (AF-100–11), 10 ng/ml FGF-2 (AF-100-18B), 5 ng/ml VEGF165 (AF-100–20), 5 ng/ml TGF-b1 (AF-100-21C; all growth factors from Peprotech), 100 U/ml penicillin, and 100 µg/ml streptomycin. EHM were left to condensate for 3 days and then transferred to a flexible maturation scaffold as previously described [38]. After 4 weeks, EHM from ACT patient and control iPSC-CMs were treated with 0.25 µM DOX for 24 h. Functional analysis of EHM was performed in organ baths in Tyrode’s solution (120 mmol/L NaCl, 1 mmol/L MgCl_2_, 0.2 mmol/L CaCl_2_, 5.4 mmol/L KCl, 22.6 mmol/L NaHCO_3_, 4.2 mmol/L NaH_2_PO_4_, 5.6 mmol/L glucose and 0.56 mmol/L ascorbic acid; gassed with 5% CO_2_/95% O_2_) at 37 °C. EHM were electrically stimulated at 1.5 Hz with 5 ms square pulses of 200 mA. EHM were preloaded to maximum isometric force generation. Spontaneous beating frequency, force of contraction was measured under gradually increasing calcium concentration (0.2–4 mM) to document maximal inotropic capacity and calcium sensitivity.

### Apoptosis assay with flow cytometry

The rate of apoptosis in our samples was observed with the APC-Annexin V Apoptosis Detection Kit with PI (BioLegend). 1.5 × 10^5^ iPSC CM were prepared according to the manufacturer's protocol.

### Analysis of sarcomeric organization

The myofilament structure was visualized by immunofluorescence staining for α-actinin. Using the software FIJI, the integrated plugin “Tubness” as well as the 2D fast Fourier transform algorithm was applied to the obtained images and resulting frequency domains were radially integrated with the open-source plugin “Radial Profile Plot” from Paul Baggethun (https://imagej.nih.gov/ij/plugins/radial-profile.html as of October 19, 2017). The software LabChart (BDInstruments) was used to automatically analyze the relative amplitude of the first peak in the intensity profile. This amplitude was used to assess the regularity of α-actinin striations in iPSC CM. To avoid bias during image acquisition, iPSC CM were selected using the DAPI channel, hence the researcher was blinded to the sarcomeric structure.

### Mito-tracker analysis

For analyzing the DOX effect on the mitochondrial area, cells were treated with 0.25 µM, 0.5 µM and 1 µM for 24 h and 0.5 µM for 72 h at 37 °C. IpSC-CM were washed twice with PBS and loaded with 250 nmol/L MitoSpy Orange CMTMRos fluorogenic mitochondrial probe (Biolegend) for 30 min at 37 °C prior to fixation. Cells were fixed using Roti-Histofix 4% solution (Carl Roth) for 20 min at RT and blocked in 1% BSA in PBS at 4 °C overnight. The mitochondrial area of interest was analyzed by FIJI plugin “Mitochondrial Network Analysis (MiNA)” toolset [41]. The branch length was calculated by the mean of all measured branches in the image. Networks were characterized by the number of objects in the image that contain at least 1 junction pixel and are thus comprised of more than one branch in percent to the whole analyzed number of objects.

### Electron microscopy analysis

IPSC-CM were fixed with 4% formaldehyde and 2.5% glutaraldehyde in 0.1 M phosphate buffer pH 7.3 and prepared for electron microscopy essentially as described [47]. In brief, the adherent cultures were postfixed on the culture dish with 1% OsO4 (Science Services, Munich, Germany) in 0.1 M phosphate buffer and embedded in Epon (Serva, Heidelberg, Germany) after dehydration with ethanol and en bloc staining with 1.5% uranyl acetate (Merck, Darmstadt, Germany) and 1.5% tungstophosphoric acid (Merck, Darmstadt, Germany) in 70% ethanol. Ultrathin sections of cultured cells were cut parallel to the substrate using an UC7 Ultramicrotome (Leica, Vienna, Austria) and stained with UranyLess® (Science Services, Munich, Germany). Sections were analyzed with an LEO EM912 Omega (Zeiss, Oberkochen, Germany) and digital micrographs were obtained with an on-axis 2048 × 2048-CCD camera (TRS, Moorenweis, Germany).

### Statistical analysis

Data is depicted as mean + standard error of the mean (SEM). For parametric data of two groups unpaired Student`s t-test was used; for non-parametric data of two groups Mann–Whitney test was used. 1- and 2-way analysis of variance (ANOVA) was applied to data sets with more than two groups. Multiple comparisons between the control and the patient group were corrected according to Sidak, whereas multiple comparisons of DOX treatment conditions with the basal condition were corrected according to Dunnett. Statistical significance is depicted as *p < 0.05, **p < 0.01, ***p < 0.001. Statistical analysis was performed with Prism 8 (GraphPad).

## Results

### Characterization of human ACT myocardium

**Fig. 1:**
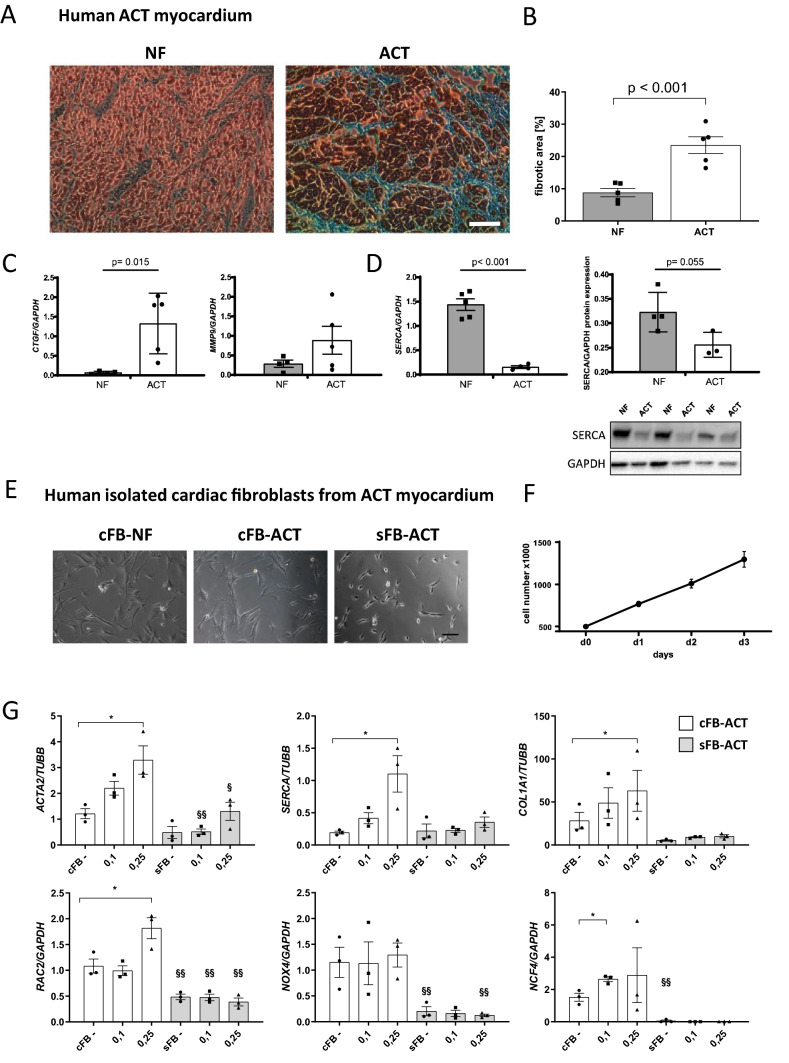
Analysis of human myocardium of ACT patients and isolated ACT cardiac fibroblasts. **A** Representative myocardium of non-failing donor (NF) and ACT patients (ACT) after trichrome staining. Scale bar: 100 µm. **B** Quantification of trichrome staining; NF: *n* = 5; ACT: *n* = 5 (biological replicates). 4 technical replicates from different tissue slices for each biological replicate were used. **C** mRNA expression of fibrosis-associated genes (*CTGF, MMP9*) in human ACT myocardium compared to NF by qPCR. Statistical analysis was performed using Student’s *t* test. *p* values: *P* > 0.05 are defined as statistically significant. Bars indicate mean values ± SEM. NF: 4–5; HF: 5. **D** mRNA and protein expression of SERCA2a. Representative Western Blots for SERCA2a in NF and ACT myocardium are shown. NF: *n* = 4; HF: *n* = 3. GAPDH was used as an internal control. Statistical analysis was performed using Student’s *t* test. *p* values: *p* > 0.05 are defined as statistically significant. Bars indicate mean values ± SEM. **E** NF cardiac fibroblasts (cFB-NF), cardiac fibroblasts from the ACT patient (cFB-ACT), and skin FB of ACT patient (sFB-ACT) show typical FB morphology. Scale bar: 100 µm. **F** Proliferation capacity over three days of human cardiac fibroblasts from 5 end-stage heart failure patients. **G** Relative DOX-dependent mRNA expression of fibrosis- and Ca^2+^-associated genes and NADPH oxidase subunits by qRT-PCR for the genes *ACTA2, COL1A1*, *SERCA*, *NOX4, RAC2,* and *NCF4* in cFB from the ACT patient (cFB-ACT) and sFB (sFB-ACT) under basal condition (−) and different DOX treatment concentrations (0.1 µM and 0.25 µM). *n* = 3 biological replicates (samples from three different passages per cell type) for each measurement. Statistical analysis was performed with 1-way ANOVA analysis or Student’s *t* test. *p < 0.05 in the cFB group and as §*p* < 0.05, §§*p* < 0.01 between cFB and sFB. Bars indicate mean values with ± SEM

To investigate the importance and severity of ACT, we analyzed samples of human explanted myocardium from five patients with end-stage heart failure that was developed after anthracycline chemotherapy and compared these to samples of healthy non-failing (NF) controls (Table 1). Fibrosis was analyzed using Masson’s trichrome staining followed by quantification of fibrotic areas in relation to myocardium. In the ACT patients, we found massive fibrosis in 24.4%, a rate significantly higher compared to healthy controls (8.5%) (Fig. 1A, B). Accordingly, there was a significantly increased expression of fibrotic markers such as connective tissue growth factor (CTGF) and matrix metalloproteinase 9 (MMP9) in the ACT patient samples (Fig. 1C). In addition, we examined the expression of SERCA2a as a key player in excitation/contraction coupling and found it to be significantly downregulated on the mRNA and protein level in ACT patients (Fig. 1D). These data confirmed that fibrosis is a significant feature of end-stage ACT myocardium.

**Table 1:** Characteristics of end-stage heart failure ACT patients used for fibrosis analysis and cardiac fibroblasts isolation

Patient	LVEF [%]	Sex	Age at TX	Cancer type	Myocardium/cardiac fibroblasts used
ACT-4	20	Female	65	Mammary carcinoma	Figure 1, S. Fig. 2
ACT-5	10–15	Male	54	Ewing sarcoma	Figure 1, S. Fig. 2
ACT-6	20	Female	53	Wilms tumor	Figure 1, S. Fig. 2
ACT-7	20	Male	16	B cell non-Hodgkin gastric lymphoma	Figure 1, S. Fig. 2
ACT-8	15	Male	43	Unknown	Figure 1, S. Fig. 2

### Characterization of isolated human cardiac fibroblasts from ACT myocardium

To investigate the hypothesis that cFB contributes together with CM to the development of ACT, cFB were isolated from the left ventricle of ACT human myocardium and characterized as to their origin. We were able to demonstrate that isolated cardiac cells have a characteristic FB morphology and a high proliferative capacity, and express typical FB genes and proteins (Fig. 1E, F; Supplementary Fig. 2A, B). DOX treatment of ACT-cFB and ACT skin FB (sFB) revealed that DOX markedly increased the expression of *ACTA2* and *COL1A1,* indicating trans-differentiation into active ACT-cFB compared to mostly unchanged expression in sFB (Fig. 1G). Interestingly, *SERCA2a* expression was enhanced upon DOX treatment in ACT-cFB, but not in sFB (Fig. 1G). We observed that certain NADPH oxidase subunits such as *RAC2* and *NCF4* increased in expression after DOX, but there was little effect on *NOX4* in both ACT-cFB and sFB. Furthermore, expression levels were markedly decreased in sFB for all tested markers compared to ACT-cFB (Fig. 1G). These results indicate that cFB isolated from human ACT myocardium exhibit an increased myofibroblastic stress response after DOX treatment.

### Recruitment of ACT patients for iPSC generation

We aimed to generate iPSC CM from ACT patients to perform a patient-specific analysis of the long-term DOX-effects in human CM. For this, we recruited five patients from the ‘rituximab with CHOP over age 60 years’ RICOVER60 trial (www.clincaltrials.gov: NCT00052936). All of these patients suffered from diffuse large B cell lymphoma and had been treated with DOX as part of a CHOP-14 treatment (cyclophosphamide, doxorubicin, vincristine and prednisolone in two-week intervals). RICOVER60 trial participants were classified into ACT patients and controls based on Reichwagen et al. [33], with symptoms such as reduced left ventricular ejection fraction (LVEF < 45%), arrhythmia, or heart failure treatment for the ACT. Based on these criteria, we recruited three patients for our study (referred to as ACT patients) who suffered from clinical cardiotoxicity (post-treatment LVEF: < 45%) as well as two patients (controls) who did not experience clinical cardiotoxicity after chemotherapy (post-treatment LVEF: > 55%) (Table 2).

**Table 2:** Characteristics of ACT patients and controls from the RICOVER60 trial used for iPSC generation

patient	Sex	Age in 2007	Median DOX treatment [mg/m^2^]	ACT	iPSC generated and used
ACT-1	Male	69	309	Chronic	Figs. 2, 3, 4, 5, 6, 7 and 8, S. Fig. 3–8
ACT-2	Male	71	309	Chronic	Figs. 2, 3, 4, 5, 6, 7 and 8, S. Fig. 3–8
ACT-3	Female	66	309	Chronic	Figs. 2, 3, 4, 5, 6, 7 and 8, S. Fig. 3–8
Control 1	Male	66	318	No	Figs. 2, 3, 4, 5, 6, 7 and 8, S. Fig. 3–8
Control 2	Male	68	318	No	Figs. 2, 3, 4, 5, 6, 7 and 8, S. Fig. 3–8

### Generation and characterization of iPSC-derived cardiomyocytes

We generated integration-free iPSC from dermal fibroblasts from three ACT patients and two controls from the RICOVER60 trial. Two independent iPSC cell lines were generated per patient and analyzed for their pluripotency. All generated iPSC maintained full pluripotency and spontaneous in vitro and in vivo differentiation capacity (Supplementary Fig. 3A–C). Both, ACT- and control-iPSC were differentiated into ventricular CM using standardized WNT modulation [24] and metabolic selection [39]. Individual batches of ventricular iPSC CM were analyzed regarding differentiation efficiency and purity. Ventricular iPSC CM displayed well-organized striated patterns visualized by α-actinin and titin immunofluorescence staining, and showed 89.70% ± 1.497% cTNT^+^ CM (Supplementary Fig. 4A, B). High expression of general cardiac markers in iPSC CM was confirmed by qPCR for *TNNT2, ACTN2, MYH6, MYH7* (Supplementary Fig. 4C)*.* Immunocytochemical staining of cardiac subtype-specific proteins showed that 94% of ventricular cells were stained positive for the ventricular isoform of myosin light chain 2 and negative for the atrial isoform of myosin light chain 2 (MLC2V + /MLC2A–) (Supplementary Fig. 4D; E). Also, ventricular differentiation was confirmed on mRNA level by qPCR of *MLC2v* and the absence of atrial markers NR2F2 and PITX2 (Supplementary Fig. 4F). Of note, proliferation was significantly decreased from 100% in iPSC to 3.0–7.5% in iPSC CM of all patients demonstrated by EdU analysis (Supplementary Fig. 5A, B). Taken together, all generated iPSC lines show full pluripotency and cardiac differentiation into predominantly ventricular iPSC CM.

### DOX resorption and retention in iPSC CM of ACT patients and controls

**Fig. 2:**
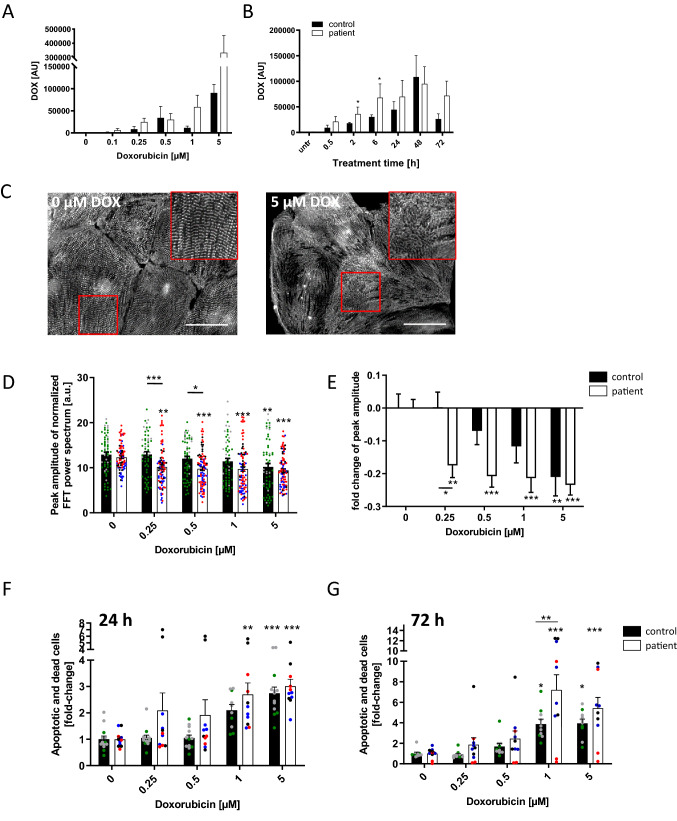
Assessment of in vitro DOX-induced cardiotoxicity in patient-specific iPSC CM. **A**, **B** DOX resorption and retention in iPSC CM. Intracellular DOX levels were investigated with HPLC in regard to DOX concentration (24 h treatment) **(A)**, treatment time (1 µM DOX) **(B)**, ACT patient: *n* = 5 differentiations from 2 patients. Control: *n* = 3 differentiations from 2 controls. **C**–**E** Effect of DOX on sarcomeric regularity in iPSC CM. **C** Immunofluorescence staining visualized α-actinin protein expression and sarcomeric organization. DOX treatment impaired sarcomeric regularity. Scale bars: 50 μm. **D**, **E** Quantification of DOX-treated (24 h) sarcomeric regularity using Fast Fourier Transform algorithm. Sample numbers: 60 control iPSC CM from 4 differentiations, 90 patient iPSC CM from 6 differentiations. **F**, **G** Effect of DOX on iPSC CM cell death. Annexin V/PI apoptosis tests showed that the number of apoptotic and dead cells rose with increasing DOX levels after 24 h (**F**) and 72 h (**G**). 10 control CM differentiations, 10–11 patient CM differentiations. Statistical analysis was performed using 2-way ANOVA analysis and Sidak’s or Dunnett’s multiple comparison, or mixed-effects analysis. Bars indicate mean values with SEM. *p < 0.05, **p < 0.01, ***p < 0.001. *Above single bars indicate statistical significances to untreated (0 µM DOX) conditions of the same group. Different colored dots indicate the different control lines (green: control 1, grey: control 2) and patient lines (blue: ACT1, black: ACT2 and red: ACT3) used

Differences in the pharmacokinetics between patients can result in vastly different efficacy or side effects. We, therefore, treated the ACT CM with increasing DOX concentrations or durations based on pharmacokinetic characteristics of DOX in humans [34], and analyzed the intracellular DOX levels in ACT CM in comparison to control CM. Both ACT CM and control CM showed a positive correlation between DOX treatment concentration and intracellular DOX levels, with consistently higher intracellular DOX levels in ACT-iPSC CM (Fig. 2A). Time-dependent DOX resorption experiments demonstrated increased intracellular DOX concentrations with peak values after 48 h of treatment (Fig. 2B). Of note, on the basis of this data as well as prior reports [4], we primarily selected the time point of 24 h (for most experiments) and used DOX at concentrations in the range of 0.1–5 µM for all further experiments in iPSC CM, cFB and Engineered Human Myocardium (EHM).

### DOX-induced cardiotoxicity is increased in ACT-iPSC CM

Direct myofibril damage can be visualized by α-actinin staining and is a hallmark of doxorubicin cardiotoxicity [15]. To assess the sarcomeric integrity after treatment with DOX, we analyzed the sarcomeric structures in ACT- and control-iPSC CM. The sarcomeric regularity was visibly impaired after treatment with 5 µM DOX for 24 h (Fig. 2C). Quantification using Fast Fourier Transformation (FFT) and radial integration confirmed the dose-dependent decrease of sarcomeric regularity in both groups (Fig. 2D). Using the clinically relevant concentration of 0.25 µM DOX, the impairment of sarcomeric regularity was significantly augmented in ACT CM compared to control CM (Fig. 2D, E, Supplementary Fig. 6A). However, 0.25 µM DOX did not reduce cell viability in control or ACT-iPSC CM (Supplementary Fig. 6B).

To analyze programmed cell death, we performed flow cytometry of iPSC CM that were positively stained for both annexin V and propidium iodide (PI). We found a dose-dependent relative increase in both apoptotic and dead cells after DOX incubation for 24 h and 72 h in both groups, suggesting that programmed cell death seems to be a principal mechanism of DOX-induced cell loss (Fig. 2F, G). All DOX concentrations tested resulted in increased amounts of apoptotic and dead cells in the ACT patient group compared to controls (Fig. 2F, G). A significant increase in apoptotic and dead cells was detected in ACT CM compared to control CM at a DOX concentration of 1 μM for 72 h (Fig. 2G; Supplementary Fig. 6C). These data suggest that DOX-treated iPSC CM recapitulate ACT disease phenotypes such as sarcomeric organizational damage and programmed cell death, as commonly described in ACT patients.

### Enhanced DOX-dependent mitochondrial damage and ROS production in ACT-iPSC CM

**Fig. 3:**
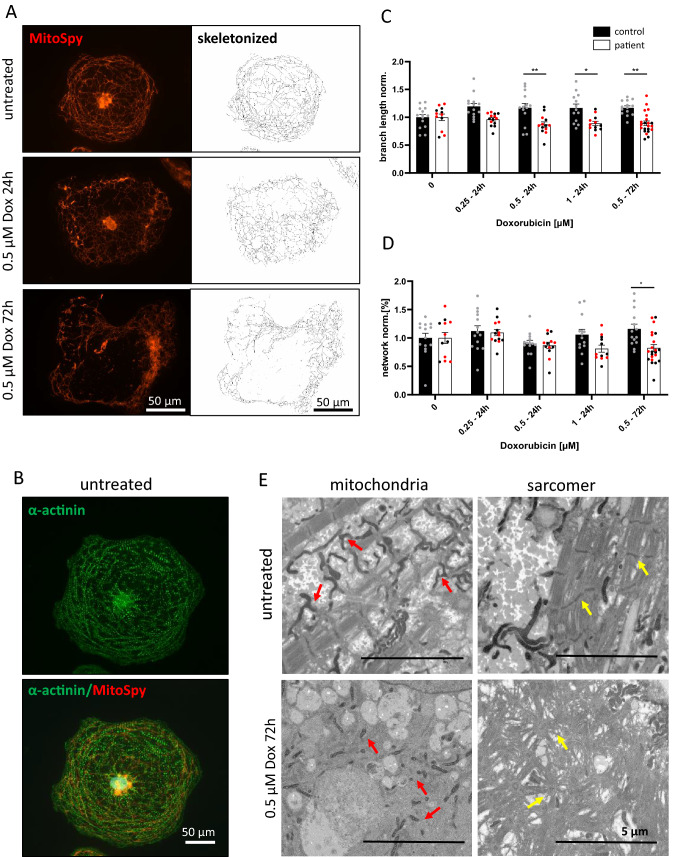
DOX-dependent mitochondrial disturbance. **A** MitoSpy staining of the mitochondrial structure under DOX (0.5 µM) treatment after 24 and 72 h in ACT-iPSC CM. The mitochondrial shape is damaged under DOX that is also demonstrated on the skeletonized stage (Mitochondrial Network Analysis software tool). Scale bars: 50 µm. **B** Example of double staining of MitoSpy (red) and cardiac marker α-actinin (green). Scale bar: 50 µm. **C**, **D** Quantification of mitochondrial branch length **(C)** and network **(D)**. The branch length and network decrease in ACT patients under DOX treatment for 24 h (0.25, 0.5 and 1 µM) and 72 h (0.5 µM). Sample numbers: 18 control-iPSC CM from 2 differentiations, 14–26 patient iPSC CM from 3 differentiations. Different colored dots indicate the different control lines (green: control 1) and patient lines (black: ACT2 and red: ACT3) used. Statistical analysis was performed using 2-way ANOVA analysis with Tukey’s multiple comparison. Bars indicate mean values with SEM. *p < 0.05, **p < 0.01. **E** Representative transmission electron micrographs of ACT-iPSC CM under DOX treatment for 72 h (0.5 µM) and untreated showing interfibrillar mitochondria (red arrows) and striated and compact sarcomers (yellow arrows) in untreated cells. After DOX treatment, the mitochondria are small and unbranched and the sarcomeric structure is irregular and disordered. Scale bars: 5 µm

A key role in DOX-dependent cardiotoxicity is attributed to damage of mitochondrial function [44]. To confirm this effect in our patient-specific ACT model, we treated control and ACT CM with different DOX concentrations (0.25 µM, 0.5 µM, or 1 µM) for 24 h or 72 h and analyzed the mitochondrial shape and network using MitoSpy. Untreated cells showed a complex and branched mitochondrial network with a regulated sarcomeric structure (Fig. 3A, B). DOX treatment subsequently disrupted the mitochondrial branch length and network in a concentration and time-dependent manner (Fig. 3A)*.* The branch length was significantly reduced in ACT iPSC CM in comparison to control cells at DOX concentrations of 0.5 and 1 µM (24 h and 72 h) (Fig. 3C). The number of mitochondrial networks in ACT patient cells was also significantly reduced in comparison to control under longer DOX treatment of 72 h (0.5 µM) (Fig. 3D). This data demonstrated that DOX impairs cardiac mitochondrial shape and network, whereas ACT CM showed significantly stronger mitochondrial damage than control CM. Ultrastructural examination of iPSC CM by transmission electron microscopy confirmed reduced branch length and reduced mitochondrial networks in DOX-treated (0.5 µM, 72 h) iPSC CM of ACT patients. Untreated iPSC CM showed longer mitochondria and a more developed branching pattern (Fig. 3E). In addition, striated and compact sarcomers become irregular and disordered after DOX treatment on an ultrastructural level confirming the immunofluorescence staining shown before (Fig. 2C–E).

**Fig. 4:**
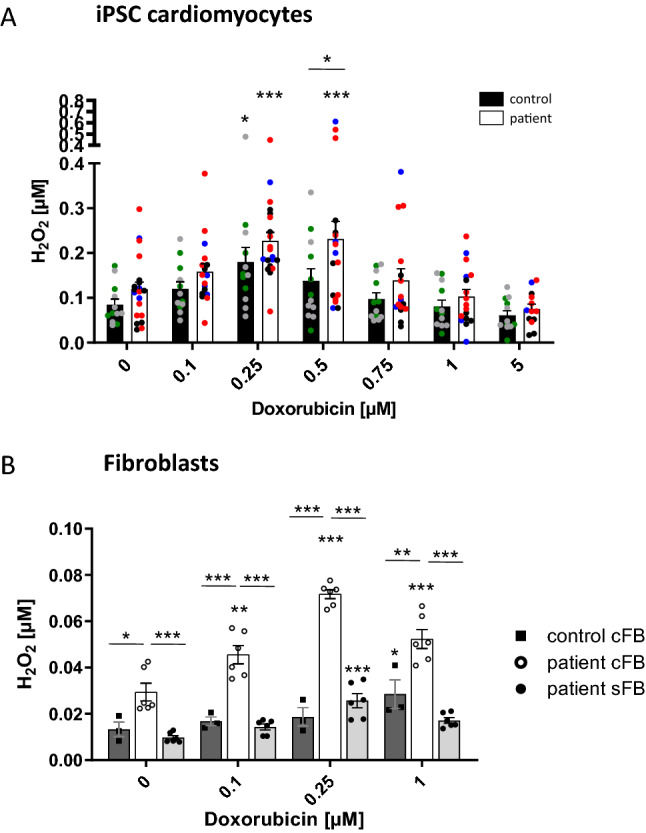
DOX-dependent ROS production in iPSC CM and cFB. **A** The amount of H_2_O_2_ in the supernatant of iPSC CM was measured with the Amplex Red assay after 24 h DOX treatment. ACT patient: *n* = 20 differentiations from 3 patients. Control: *n* = 12 differentiations from 2 controls. Different colored dots indicate the different control lines (green: control 1, grey: control 2) and patient lines (blue: ACT1, black: ACT2 and red: ACT3) used. **B** H_2_O_2_ in the supernatant of ACT patient and control cFB, and patient skin FB (patient sFB) was measured with the Amplex Red assay after 24 h DOX treatment. *n* = 3 biological replicates (samples of 3 different passages were measured of control cFB, patient cFB, and patient sFB. Statistical analysis was performed using 2-way ANOVA analysis and Sidak’s or Dunnett’s multiple comparison test. Data are shown as mean with SEM. *p < 0.05, **p < 0.01, ***p < 0.001. *Above single bars indicate statistical significances to untreated (0 µM DOX) conditions of the same group

Since mitochondria are described as one of the major sources of increased ROS production, we aimed to assess the amount of ROS in ACT-iPSC CM compared to controls by measuring extracellular H_2_O_2_ with Amplex Red. After 24 h of DOX treatment, H_2_O_2_ increased DOX-dependently with significantly higher H_2_O_2_ at 0.5 µM DOX in ACT-iPSC CM compared to control cells (Fig. 4A, Supplementary Fig. 6D). Physiological DOX concentration of 0.25 µM resulted in significantly increased H_2_O_2_ in both, control- and ACT-iPSC CM compared to untreated cells but did not influence cell viability (Fig. 4A, Supplementary Fig. 6B). Higher DOX concentrations do not influence H_2_O_2_ levels in ACT- or control-iPSC CM. Since we found a fibrotic phenotype of ACT myocardium, we furthermore analyzed DOX-induced ROS production in ACT cFB, ACT sFB and healthy control cFB. We found a DOX-dependent H_2_O_2_ increase in all tested FBs with significantly higher H_2_O_2_ amounts at all measured DOX concentrations in ACT cFB compared to healthy cFB or patient sFB (Fig. 4B). Taken together, both ACT-iPSC CM and ACT cFB showed dose-dependent increases in extracellular H_2_O_2_ compared to healthy controls, suggesting a contribution of both cell types to the development of ACT.

### Decreased DOX-dependent contractile function in ACT patients

**Fig. 5:**
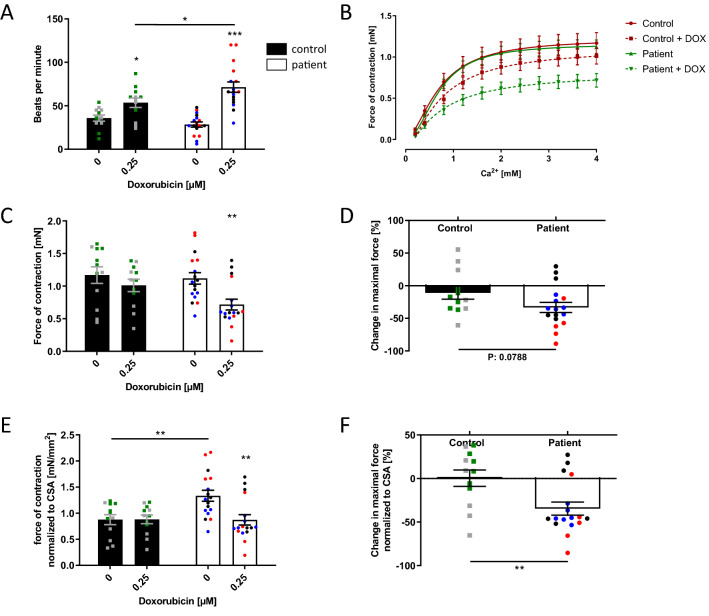
DOX decreases the force of contraction in engineered human myocardium (EHM). **A** Beating frequency of control and ACT patient EHM increases after DOX treatment (0.25 µM). **B** The generation of the contractile force of control and ACT patient EHM is Ca^2+^-dependent, and impeded by DOX treatment (0.25 µM). **C** Maximal force of contraction of control and ACT patient EHM is lowered after incubation with 0.25 µM DOX. **D** The DOX-induced relative change of maximal contractile force is greater in ACT patient EHM compared to control EHM. **E** Maximal force of contraction of control EHM and ACT patient EHM normalized to cross-sectional area (CSA). **F** DOX-induced change in the maximal force of control and ACT patient EHM normalized to CSA. **A**–**F** Sample numbers: 12 control EHM from 2 differentiations, 17 ACT patient-EHM from 3 differentiations. Different colored dots indicate the different control lines (green: control 1, grey: control 2) and patient lines (blue: ACT1, black: ACT2 and red: ACT3) used. Statistical analysis was performed using Student’s *t* test or 2-way ANOVA analysis and Sidak’s multiple comparison test. Mean with SEM. *p < 0.05, **p < 0.01, ***p < 0.001. *Above single bars indicate statistical significances to untreated (0 µM DOX) conditions of the same group

To investigate how the DOX-dependent alterations in sarcomeric integrity, ROS production, and apoptosis affect muscle function, we generated EHM from ACT or control-iPSC CM. EHM from ACT-iPSC CM and control-iPSC CM exhibited comparable spontaneous beating frequencies (control: 35.8 ± 3.5, ACT: 28.4 ± 2.9 bpm). DOX treatment induced an increase in beating frequency, which was significantly more pronounced in ACT EHM (Fig. 5A, Supplementary Fig. 7A) compared to controls. About 15% of generated EHM displayed irregular beating at basal conditions and DOX treatment increased irregular beating rate by 40% in both groups (Supplementary Fig. 7B). Furthermore, EHM from both groups showed a similar maximal force of contraction at increasing Ca^2+^ concentrations (Fig. 5B). Upon DOX treatment, the force of contraction was significantly decreased in the ACT patient group but not in the control group (Fig. 5B–D; Supplementary Fig. 7C). Despite the significant decrease in cross-sectional area (CSA) of ACT EHM compared to controls (Supplementary Fig. 7D), we still found a significant DOX-dependent decrease in the relative force of contraction in ACT EHM (by about 35%) after normalization to CSA (Fig. 5E, F; Supplementary Fig. 7E). Taken together, EHM from ACT-iPSC CM showed an increased DOX-dependent beating frequency and a decreased DOX-dependent relative force generation compared to control-iPSC CM.

### Disturbed functionality of calcium homeostasis in patients with ACT after DOX

**Fig. 6:**
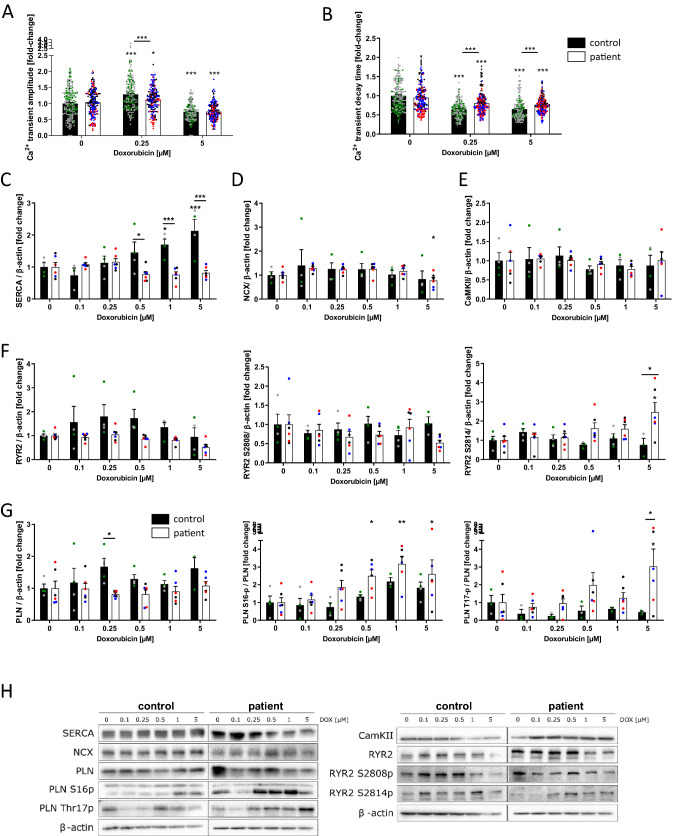
DOX disturbs Ca^2+^ homeostasis. **A**, **B** Effects of DOX (0.25 µM and 5 µM) on Ca^2+^ transient amplitude (**A**) and Ca^2+^ transient decay time (**B**) in iPSC CM of control and ACT patients. **C**–**G** DOX-induced relative changes in the amount of protein expression and phosphorylation of SERCA2a (**C**), NCX (**D**), CaMKIIδ (**E**), RYR2, RYR2-S2808, RYR2-S2814 (**F**), PLN, PLN-S16p, PLN-T17p (**G**) in iPSC CM of control and ACT patients after 24 h of DOX treatment. Different colored dots indicate the different control lines (green: control 1, grey: control 2) and patient lines (blue: ACT1, black: ACT2 and red: ACT3) used. **H** Representative Western blots of Ca^2+^-associated proteins in DOX-treated iPSC CM of control and ACT patients. Sample numbers: **A**, **B** 180 control-iPSC CM from 9 differentiations from 2 controls, 180 ACT patient-iPSC CM from 9 differentiations from 3 patients. **C**–**G** 4 control-iPSC CM differentiations, 6 ACT patient iPSC CM differentiations. Statistical analysis was performed using 2-way ANOVA with Dunnett’s multiple comparison test. Mean with SEM. *p < 0.05, **p < 0.01, ***p < 0.001. *Above single bars indicate statistical significances to untreated (0 µM DOX) conditions of the same group

Since Ca^2+^ homeostasis is the main regulator of cardiac contractile function, we investigated cellular Ca^2+^ kinetics in iPSC CM using confocal microscopy measurements (Fluo-4 calcium dye) at a therapeutic DOX dose of 0.25 µM as well as a toxic DOX dose of 5 µM. After 24 h of DOX treatment, ACT-iPSC CM exhibited a significantly reduced Ca^2+^ transient amplitude (Ca^2+^_i_) compared to control-iPSC CM at DOX concentrations of 0.25 µmol/l. DOX concentrations of 5 µM lead to decreased Ca^2+^ amplitudes in both groups (Fig. 6A). Furthermore, DOX treatment resulted in an acceleration of Ca^2+^ T50 transient decay time in both, control and ACT lines, whereas the ACT-iPSC CM exhibited significantly decelerated T50 transient decay time compared to control at DOX concentrations of 0.25 µM and 5 µM (Fig. 6B) suggesting a prolonged cytosolic Ca^2+^ elimination in the ACT.

To elucidate the underlying mechanisms of dysregulated EC coupling in iPSC CM of ACT patients and control, Western blots were performed. SERCA protein expression was found to be significantly increased in control in a DOX dose-dependent manner (0.5–5 µM) (Fig. 6C, H). In contrast, activated CaMKIIδ was shown in ACT-iPSC CM immediately leading to hyperphosphorylation of PLN-T17 as well as RYR2-S2814 upon 24 h of 0.5 µM up to 5 µM DOX treatment (Fig. 6F, G). Consequently, both conditions resulted in altered Ca^2+^ kinetics with significantly decelerated Ca^2+^ decay time in the ACT group. The expression of ryanodine receptor 2 (RYR2) was slightly, but not significantly increased upon DOX treatment in control cells, but not in ACT-iPSC CM (Fig. 6F, H). Protein expression of the Na^+^/Ca^2+^ exchanger (NCX), phospholamban (PLN), and CaMKIIδ were not regulated after DOX treatment in both groups (Fig. 6D, E, G). However, PKA-specific phosphorylation of PLN-S16 increased significantly in a DOX dose-dependent manner, predominantly in ACT-iPSC CM (Fig. 6G, H). In contrast, PKA-specific phosphorylation of RYR-S2808 did not result in DOX-induced changes (Fig. 6F, H). We also quantified the basal protein expression of calcium-handling proteins, and detected no significant differences in the amounts of SERCA2a, NCX1, PLN, CaMKIIδ, and RYR2. In addition, the phosphorylation of PLN-S16 or -T17 and RYR2-S2808 or -S2814 were similar under basal conditions in control and ACT-iPSC CM (Supplementary Fig. 8).

In summary, this data shows on a patient-specific level that DOX-dependent ROS production and subsequent hyperactivation of CaMKIIδ is limited to ACT patients, whereas control cells do not upregulate CaMKIIδ activity, but significantly increase SERCA2a expression in a DOX-dependent manner to avoid heart failure conditions. As a functional consequence, fine-tuned differences between patient and control cells such as reduced ventricular Ca^2+^ transient amplitude in ACT- iPSC CM as well as decelerated Ca^2+^ transient decay time compared to control were demonstrated.

### DOX increases the diastolic sarcoplasmic reticulum Ca^2+^ leak and proarrhythmic triggers

**Fig. 7:**
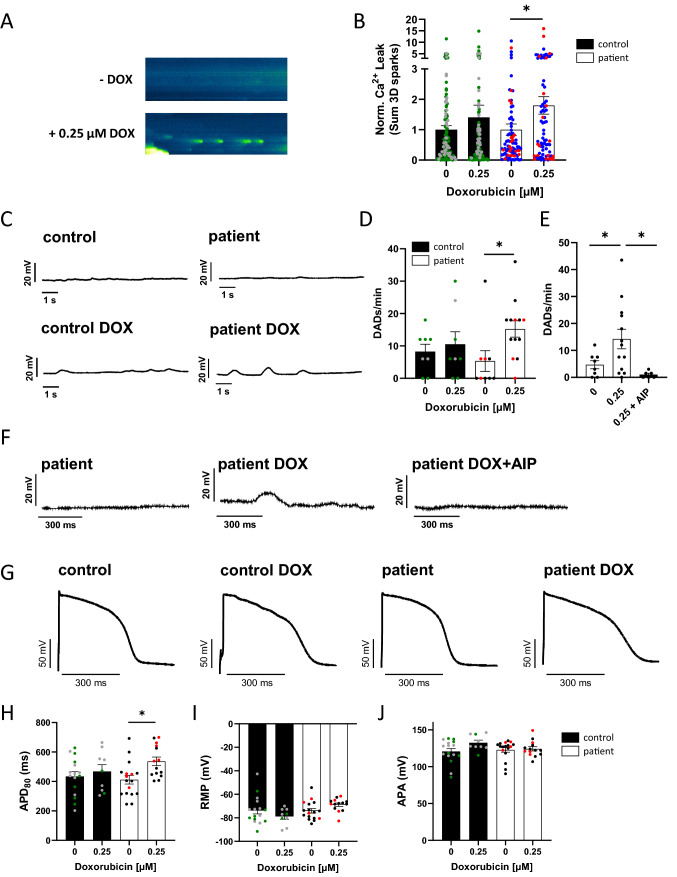
Effects of DOX on cellular electrophysiology and proarrhythmic triggers. **A** Representative confocal line scans (Fluo-4) showing diastolic sarcoplasmic reticulum Ca^2+^ sparks and **B** mean sarcoplasmic reticulum Ca^2+^ leak (norm. to untreated) after DOX treatment (0.25 µM, 24 h) in ACT patients (untreated (0 DOX): *n* = 79 cardiomyocytes /4 differentiations; 0.25 µM DOX: *n* = 79/4) versus control (untreated (0 DOX): *n* = 120/6; 0.25 µM DOX: *n* = 120/6). *p* values were calculated using Mann–Whitney test. **C** Original unstimulated recordings (whole-cell current-clamp) of iPSC CM during 10 s rest after a series of 30 elicited action potentials at 1 Hz showing delayed afterdepolarizations (DADs) after treated with DOX for 24 h. **D** Incidence of DADs/min of iPSC CM after DOX treatment for 24 h in patients (Untreated: *n* = 9 cells/3 differentiations; DOX: *n* = 13/3) compared to control (untreated: *n* = 8/2; DOX: *n* = 8/2). **E** Incidence of DADs/min of iPSC CM after DOX treatment for 24 h in patients (Untreated: *n* = 8 cells/2 differentiations; DOX: *n* = 13/2) compared to iPSC-CM from patients treated with DOX (0.25 µM) and AIP (1 µM), both for 24 h (*n* = 8/2; DOX: *n* = 6/2). **F** Original recordings (whole-cell current-clamp) of DADs during rest after stimulated action potentials in iPSC CM following treatment with 0.25 µM DOX for 24 h, which were abolished by CaMKII-Inhibitor AIP (24 h, 1 µM). **G** Representative original action potential recordings (whole-cell current-clamp) of iPSC CM after DOX treatment (24 h) in control compared with ACT patients. **H** Mean values for APD80, **I** RMP and **J** APA after DOX treatment for 24 h in patients (Untreated: *n* = 17 /3; DOX: *n* = 14/3) compared to control (untreated: *n* = 16/2; DOX: *n* = 9/2). Different colored dots indicate the different control lines (green: control 1, grey: control 2) and patient lines (blue: ACT1, black: ACT2 and red: ACT3) used. **A** *p* values were calculated using Kruskal Wallis test (**D**, **H**–**J**) or Mann–Whitney test (**B**)

As RyR2 was found to be hyperphosphorylated in ACT patients upon DOX, we investigated the proarrhythmic diastolic sarcoplasmic reticulum (SR) Ca^2+^ leak. We performed confocal microscopy of ACT- and control-iPSC CM upon DOX treatment for 24 h to examine diastolic SR Ca^2+^ sparks. As demonstrated in Fig. 7A/B, we detected a significantly increased diastolic SR Ca^2+^ leak in ACT patients upon DOX treatment compared to healthy controls. To investigate whether an increased diastolic SR Ca^2+^ leak may initiate proarrhythmic cellular triggers in iPSC CM of ACT patients, we performed patch-clamp experiments (whole-cell current-clamp) so as to investigate the occurrence of delayed afterdepolarizations (DADs). As demonstrated in Fig. 7C/D, no significant changes in the DAD incidence in ACT patients under basal conditions compared to controls were found. Interestingly, DOX significantly increased the DAD incidence in iPSC CM of ACT patients. To test the causal contribution of CaMKII for the DOX-induced DAD incidence in ACT-iPSC CM, simultaneous treatment of iPSC CM using DOX (0.25 µM) and the CaMKII-inhibitor autocamtide-2-related inhibitory peptide (AIP, 1 µmol/l) was performed for 24 h. AIP reduced the increased DAD incidence in patient’s iPSC-CM and, therefore, rescues the proarrhythmic cellular triggers (Fig. 7E/F) confirming the causal contribution of CaMKII to the DOX-dependent development of arrhythmia. Electrophysiological studies were performed to clarify whether ACT might be associated with further electrical remodelling. In patch-clamp experiments of ACT- and control-iPSC CM, we observed a significantly prolonged action potential duration (APD_80_) in ACT patients compared to control-iPSC CM after 24 h DOX treatment (Fig. 7G, H), whereas resting membrane potential (RMP) or action potential amplitude (APA) were not altered (Fig. 7I, J). Untreated ACT patients showed no change of the action potential, APD, RMP or APA compared to control CM (Fig. 7G–J). The prolongation of the APD may cause additional arrhythmogenic triggers such as early afterdepolarizations (EADs) and alterations in repolarization but also contractile dysfunction as typical hallmarks of electrophysiological remodelling in heart failure.

### Effect of DOX on patient-specific gene expression

**Fig. 8:**
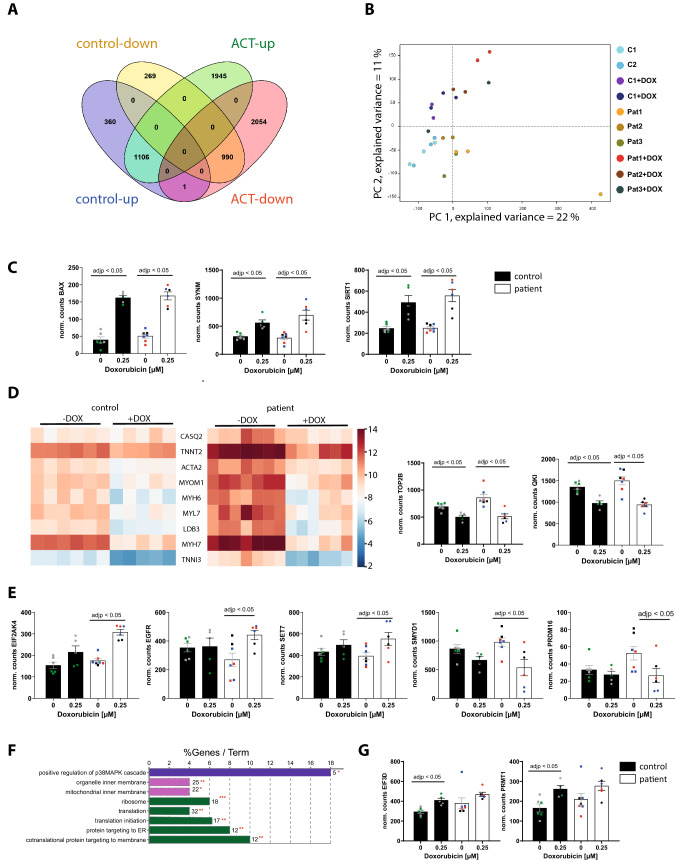
DOX-dependent differential gene expression in both ACT patient EHM and control EHM. **A**–**F** For control EHM, 5–6 independent EHM from 1 cell line of control patient 1 and 2, and for ACT patient EHM, 6–7 independent EHM from 1 cell line of each of the 3 ACT patients (ACT1, ACT2, ACT3) were analyzed. **A** DOX-induced differentially expressed genes (DEGs) in ACT patient EHM and control EHM compared to untreated conditions illustrated in a Venn diagram. **B** PCA plot of control and ACT patient EHM samples used for the analysis. **C** Normalized counts of DOX-dependent upregulated DEGs (*BAX, SYNM, SIRT1*). **D** Heat map of 9 structural genes that are significantly downregulated after DOX treatment in control and ACT patient EHM. Normalized counts of DOX-dependent downregulated TOP2B and QKI. **E** Normalized counts of ACT-specific, DOX-dependent upregulated DEGs (*SET7, EIF2AK4, EGFR*) as well as downregulated DEGs (*SMYD1, PRDM16*). **F** Significant enriched GO terms after DOX in control EHM compared to untreated conditions according to ClueGo Cytoscape plugin. The data of differential expression are based on all up-regulated genes. Data are generated by GO cellular component, GO biological process or KEGG pathway analysis. Terms of the same color correspond to terms containing a similar group of genes. The bars represent the number of the genes from the analyzed cluster that were found to be associated with the term, and the label displayed on the bars is the percentage of genes found compared to all the genes associated with the term. *p < 0.1; **p < 0.05 calculated based on hypergeometric distribution from Database for Annotation, Visualization and Integrated Discovery (DAVID, v6.7). **G** Normalized counts of control-specific, DOX-dependent upregulated DEGs (*EIF3D, PRMT1)*

To further elucidate and confirm the molecular mechanisms underlying the pathogenesis of the DOX-induced phenotype, we performed RNA sequencing of EHM derived from three ACT patients and two controls, both with and without 0.25 µM DOX exposure for 24 h. We were able to identify the differentially regulated genes (DEGs) between the untreated EHM population and the EHM after DOX treatment (Fig. 8A). After normalization for baseline expression, we identified 1106 upregulated DEGs and 990 downregulated DEGs in both ACT patient and control after DOX (Fig. 8A). Principle component analysis (PCA) of the ACT and control groups treated and not treated with DOX showed a low variation within the groups but allowed a clear distinction due to high variation between −/ + DOX (Fig. 8B). GO enrichment analysis of DOX-induced DEGs in both control and ACT EHM identified significantly increased expression of genes with key functions including programmed cell death (*BAX*), or epigenetic enzymes (histone deacetylase *SIRT1*) (Fig. 8C, Supplementary Table 1). Furthermore, we found significant decreases in actin cytoskeletal organization (18.03%, e.g. *ACTA2, MYH6, MYH7, MYL2, MYL7, TNNI3, TNNT2*), muscle contraction (8.74%, e.g. *LDB3, MYH6, MYOM1, TNNT2*), and chromosome organization (*TOP2B*) in both groups after DOX treatment (Fig. 8D, and Supplementary Table 2). The downregulation of the RNA binding protein *QKI* after DOX-treatment in control and ACT EHM was confirmed, as described in our previous study for rodent CMs and human iPSC CM (Fig. 8D) [15]. The downregulation of cardiac structural genes in response to DOX is illustrated in a heat map for control and ACT patients (Fig. 8D) and was also established in an animal in vitro and in vivo model [19]. We confirmed and validated a consistent downregulation of transcripts such as α-actinin *(ACTN2)*, cardiac troponin T *(TNNT2),* α-myosin heavy chain *(MYH6)*, and β-myosin heavy chain *(MYH7)* after DOX treatment by qPCR in human 2D iPSC CM in both populations (data not shown). These data suggest that DOX-treated EHM recapitulates general ACT disease mechanisms such as programmed cell death, myofibril damage or catabolic processes, and confirm the increased DOX-dependent apoptosis and sarcomeric dysregularity in iPSC CM of control and ACT patients (Fig. 2D–H).

1945 upregulated and 2054 downregulated genes were found only in ACT patient EHM after DOX treatment (Fig. 8A, Supplementary Table 5). The upregulated genes were involved in negative regulation of protein synthesis (Eukaryotic Translation Initiation Factor 2 Alpha Kinase 4 (*EIF2AK4*)) or important epigenetic players as the histone lysine methyltransferases *SET7* and *SMYD1* (Fig. 8E, Supplementary Table 4). Of note, PR domain containing 16 (PRDM16), a repressor of TGFβ signaling and known genetic cause of left ventricular, non-compaction cardiomyopathy (LVNC) and DCM [1], was significantly downregulated in ACT iPSC CM after DOX, but not in DOX-treated control cells (Fig. 8E). Normalized counts of ACT-specific DOX-dependent DEGs are shown in Fig. 8E. These data indicate differentially regulated mechanisms such as mRNA translational processes in DOX-treated ACT EHM compared to control. Interestingly, GO-term analysis of control DOX-induced DEGs identified significantly increased expression of translation, translational initiation (*EIF3D, EIF6*), ribosome assembly (*EFNA1*), and positive regulation of p38MAPK cascade (*PRMT1*) (Fig. 8F, Supplementary Table 3). Normalized counts of *EIF3D* and *PRMT1* are illustrated in Fig. 8G.

### Genetic-based cardiac dysfunction in patients with ACT

The high inter-individual variance in ACT manifestation might be due to genetic predisposition. To test this, we performed whole-exome sequencing (WES) on DNA from skin fibroblasts of three ACT patients and two controls used in this study for the iPSC generation. Based on the hypothesis of a genetic predisposing variant with a strong functional effect, we focused our WES data analysis on the 81 genes previously associated with various genetic forms of cardiomyopathies and cardiac arrhythmias. We found that patient ACT-2 carries the heterozygous c.C2633T variant in *PRDM16* (p.P878L) that was predicted to be deleterious or damaging by different computational prediction programs. This variant does not represent a common single nucleotide polymorphism (SNP) and is not listed in over 121,000 alleles in the ExAC database. Causative variants in *PRDM16* are associated with LVNC and DCM [1], and therefore the PRDM16 c.C2633T variant from our study might be associated with ACT subject cardiotoxicity. Patient ACT-3 was found to carry several variants including the heterozygous rare variant c.3010G > A (V1004I) (present in 2 of < 121,000 in the ExAC) in Synemin (*SYNM*), and the heterozygous rare variant c.82924G > A (p.V27642M) in TTN. Furthermore, patient ACT-3 harbors the variant c.2601del (p.E868Nfs*35), which causes a frameshift in the Epidermal Growth Factor Receptor (EGFR). No likely candidate variant was found in the other ACT-1 patient. All variants are sown in Supplementary Table 9.

## Discussion

In the present study, we showed for the first time that human iPSC CM and EHM can recapitulate a B cell lymphoma patient’s susceptibility to DOX-induced cardiac dysfunction when exposed to clinically high dosages of DOX. ACT-iPSC CM are consistently more sensitive to DOX toxicity and have demonstrated increased disorganized myofilament structure, changed mitochondrial shape, increased cell death, disturbed Ca^2+^ cycling and electrophysiology. An increase in DOX-dependent ROS production was identified in ACT-iPSC CM, as well as in ACT cFB from strong fibrotic human left ventricular ACT myocardium. All these processes are supported by transcriptome analysis of EHM, revealing significant general DOX-dependent gene expression changes in pathways related to “programmed cell death”, “ROS production”, or “striated muscle contraction” in both groups, that is in line with recently published studies of DOX-induced general changes in gene expression [21, 43]. We were also able to demonstrate that ACT-iPSC CM are not able to adapt to acute DOX stress by increasing the expression of important Ca^2+^ handling proteins such as SERCA2a to avoid heart failure conditions, as it was shown in control-iPSC CM. Instead, a DOX-dependent overactive CaMKIIδ with increased diastolic sarcoplasmic reticulum Ca^2+^ leak resulting in an increased DAD incidence was identified in ACT CM, suggesting a cellular link to arrhythmogenic events and contractile dysfunction found in ACT EHM. We found precise differences between control and ACT groups on a cellular, molecular and functional level, confirming the suitability of our patient-specific model system and opening the possibility to study the drug susceptibility of high-risk ACT patients.

Our study adds fundamental knowledge to recent reports demonstrating the use of iPSCs from breast cancer patients to recapitulate their predilection to ACT [4, 21]. To our knowledge, ours is the first human iPSC CM model of DOX-induced cardiac dysfunction in patients with aggressive B cell lymphoma who were treated with high doses of DOX. In our model, the ACT phenotype can not only be studied in a 2D monolayer of iPSC CM but in a myocardium-like, more mature human 3D EHM culture [38], allowing for a functional and morphological comparison to the myocardium of end-stage heart failure ACT patients. Reduced ventricular ejection fraction and myocardial contractile dysfunction are characteristic of patients with DOX-induced cardiac dysfunction as well as dilated cardiomyopathy [6]. We demonstrated a decreased force of contraction in DOX-treated ACT EHM, similar to clinical features of ACT patients and to iPSC EHM of patients with DCM [36]. In addition, we demonstrated a DOX-dependent increased beating frequency in ACT EHM that may mimic potential rhythm disturbances in patients with acute ACT immediately after DOX treatment [6].

**Fig. 9:**
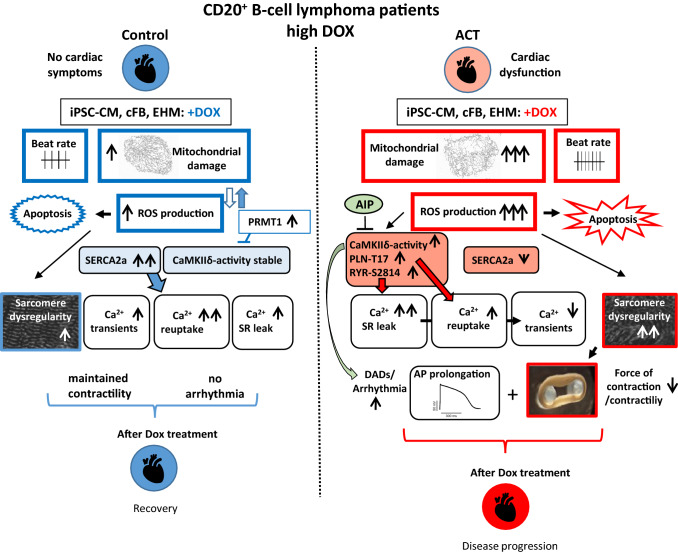
Individuals with CD20 + B-cell lymphoma who had received high doses of DOX and suffered cardiac dysfunction were studied and compared to control-iPSC CM from cancer survivors without cardiac symptoms. In cellular studies, ACT-iPSC CM were persistently more susceptible to DOX toxicity including augmented, mitochondrial dysregulation, increased reactive oxygen species, increased apoptotic events, higher beating rate, and disorganized myofilament structure. ACT cells exhibited hyperactive CaMKIIδ and hyperphosphorylation of PLN-T17 and RYR2-S2814 but lowered SERCA2a expression. Ca^2+^ transient amplitude of ACT-iPSC CM was reduced compared to control cells, and diastolic sarcoplasmic reticulum Ca^2+^ leak was DOX-dependently increased. Prolonged action potentials in ACT CM suggest a cellular link to arrhythmogenic events and contractile dysfunction especially found in ACT engineered human myocardium. The CaMKII-inhibitor AIP rescues the DAD incidence in ACT-iPSC CM, confirming the contribution of the CaMKII to DOX-dependent altered electrophysiology, proarrhythmic triggers and ACT development. In contrast, control CM upregulated SERCA2a expression in a DOX-dependent manner, without altering CaMKIIδ activity. Therefore, control cancer survivors recover after DOX treatment without cardiac symptoms, whereas patients persue cardiac disease progression

Furthermore, our patient-specific data provide new mechanistic concepts on how DOX might impair cardiac function by modulating the activity or expression of important Ca^2+^ handling proteins leading to altered CM Ca^2+^ function. Since augmented mitochondrial ROS production is a major trigger for ACT, downstream effects of elevated ROS were analyzed including the oxidation and thereby activation of proteins such as CaMKIIδ [11]. In molecular investigations, we found hyperphosphorylated PLN-T17 as well as RYR2-S2814 especially in ACT CM upon DOX treatment (Fig. 6F/G) likely caused by activated CaMKIIδ. This is in line with previous studies in neonatal and adult rat myocytes demonstrating a DOX-dependent overactive CaMKIIδ [35]. Accordingly, the open probability of the RYR2 is increased [46], leading to a significantly augmented diastolic sarcoplasmic reticulum Ca^2+^ leak as demonstrated in DOX-treated ACT CM (Fig. 7A/B). Control cells do not upregulate CaMKIIδ activity, but significantly increase SERCA2a expression in a DOX concentration-dependent manner as a compensatory mechanism to avoid Ca^2+^ overload. As a consequence, treatment with DOX leads to acceleration of Ca^2+^ cycling in control and ACT lines, whereas the ACT lines exhibited significantly slower cytosolic Ca^2+^ elimination after DOX (Fig. 6B). This is in line with the frequently reported decreased DOX-dependent or independent SERCA2a activity in heart failure myocardium in the present (Fig. 1D) and in previous studies [52]; which is functionally manifested in lower Ca^2+^ reuptake into the SR and cytoplasmic Ca^2+^ overload. The combination of both, RyR2 mediated Ca^2+^ leak and decreased SERCA expression, likely explains the reduced ventricular Ca^2+^ transient amplitude in ACT-iPSC CM compared to control (Fig. 9). Electrophysiological studies demonstrated an association of ACT with DOX-dependent prolonged action potentials and increased DAD events, which may induce additional arrhythmogenic triggers but also contractile dysfunction as typical hallmarks of heart failure as shown in this and other studies [40]. The contribution of CaMKIIδ to proarrhythmic triggers could be confirmed as the DOX-induced increased DAD incidence was ameliorated by CaMKII inhibition in ACT-iPSC CM. Thus, the DOX-dependent increase in CaMKII activity in combination with decreased SERCA expression may serve as an explanation for the contractile dysfunction and disease progression in ACT patients.

Therapeutic targeting of CaMKIIδ was demonstrated to improve contractile dysfunction of the failing heart in a preclinical mouse model [22]. Interestingly, our transcriptome analysis revealed that protein arginine methyltransferase 1 (PRMT1), which is essential for preventing cardiac CaMKIIδ hyperactivation and therefore pathological responses [30], is specifically upregulated in control EHM upon DOX treatment but not in the ACT EHM (Figs. 8G, 9). Whether PRMT1 activation can protect against DOX-induced heart failure has to be proven in future studies.

We and others suggest that ACT patients may possess combinations of genetic variants associated with increased susceptibility to DOX-induced cardiac dysfunction [13]. For that reason, we performed WES and identified highly relevant variants in cardiac and cancer genes, such as PRDM16 (p.P878L) in ACT patient 2, and SYNM (p.V1004I), TTN (p.V27642M), and an EGFR (p.E868Nfs*35) variant in ACT-3. While none of these specific variants have been described as pathogenic, *PRDM16, SYNM* and *TTN* are well-known cardiomyopathy-associated genes [1, 14, 51]. Furthermore, Prdm16 was shown to promote stem cell maintenance in multiple tissues, partly by regulating oxidative stress [9]. Of note, *PRDM16, SYNM*, and *EGFR* are all differentially expressed in the EHM transcriptome data of this study. While *PRDM16* and *EGFR* are specifically altered in expression in ACT EHM after DOX (Fig. 6E), *SYNM* seems to be a general DOX target since it is significantly upregulated in both ACT and control after DOX (Fig. 8C). To prove the hypothesis of a genetic predisposition to ACT, further WES experiments in larger patient cohorts will be necessary. Nevertheless, in 2 of 3 patients, we identified variants in important cardiac proteins, which are associated with cardiac pathologies. Recently, it was shown that the patient-specific iPSC model is ideal for studying the implication of transporter inhibitors and genetic variants in ACT [26].

## Limitations

It seems also important to point to the limitations of our study. First, we used two different iPS cell lines per ACT patient and control, all generated by non-integrative methods, for a minimum of 3 cardiac differentiation experiments. The maturity of iPSC CM as well as the patient’s genetic background, age and sex, may have influenced the results. In future studies, age-matched control iPSCs without previous DOX treatment should serve as ideal controls. Secondly, compared with isolated adult cardiomyocytes from the human myocardium, iPSC CM resemble an immature/neonatal status of cardiac cells. To overcome the limitation of inherently immature iPSC-derivatives in the study of late-onset diseases, we used a prolonged culture of at least 60–90 d to foster maturation, as shown in our previous study for the metabolic profile of iPSC-CM [10]. Nevertheless, the iPSC CM still does not resemble an adult phenotype. Thirdly, as the functional consequences of the failed adaptation to DOX stress in ACT-iPSC CM by modulating expression or activity of Ca^2+^ handling protein SERCA2a is still unclear, detailed functional Ca^2+^ measurements, including a comprehensive analysis of SR Ca^2+^ content, and SERCA function will be required in the future. Lastly, our data suggest the contribution of novel variants in cardiomyopathy and cancer-associated genes to the observed ACT phenotype, but the mechanisms are unclear. Therefore, detailed functional analyses including genome editing work need to be done in the future. Finally, aside from the studied DOX effects, further analyses of potential cardio-protective mechanisms and therapeutic substances, including ranolazine and dexrazoxan, need to be investigated.

## Conclusion

In conclusion, we used cFB, iPSC CM and EHM from healthy controls and ACT patients with cardiac dysfunction to demonstrate that all cell types can recapitulate a patient’s susceptibility to ACT after exposure to DOX. The iPSC CM showed characteristics comparable to mechanical dysfunctional and strong fibrotic human cardiac tissue from ACT patients with end-stage heart failure. Our results suggest that DOX-dependent altered Ca^2+^ signaling on a patient-specific level underlie CM dysfunction. Targeting the Ca^2+^ signaling pathway by SERCA2a or CaMKIIδ inhibition may thus represent a new approach for mitigating the cardiac side effects of DOX in cancer patients. Our data show that the patient-specific stem cell platform of ACT is of high importance to unravel the underlying signaling mechanism and to identify genetic variants associated with ACT providing also new targets for the therapy of ACT.

## Supplementary Information

Below is the link to the electronic supplementary material.

Supplementary file1 (PDF 688 KB)

Supplementary file2 (XLS 146 KB)

Supplementary file3 (XLS 39 KB)

Supplementary file4 (XLS 35 KB)

Supplementary file5 (XLS 29 KB)

Supplementary file6 (XLS 19 KB)

Supplementary file7 (DOCX 74 KB)

Supplementary file8 (XLSX 159 KB)

Supplementary file9 (PDF 878 KB)

Supplementary file10 (PDF 309 KB)
