# Supplementary material for: Doxorubicin induces cardiotoxicity in a pluripotent stem cell model of aggressive B cell lymphoma cancer patients
Source: Basic Res Cardiol. 2022 Mar 8;117(1):13. doi: 10.1007/s00395-022-00918-7 (PMC8904375; doi:10.1007/s00395-022-00918-7)

ACT-patient after DOX treatment, Fig. 7 I

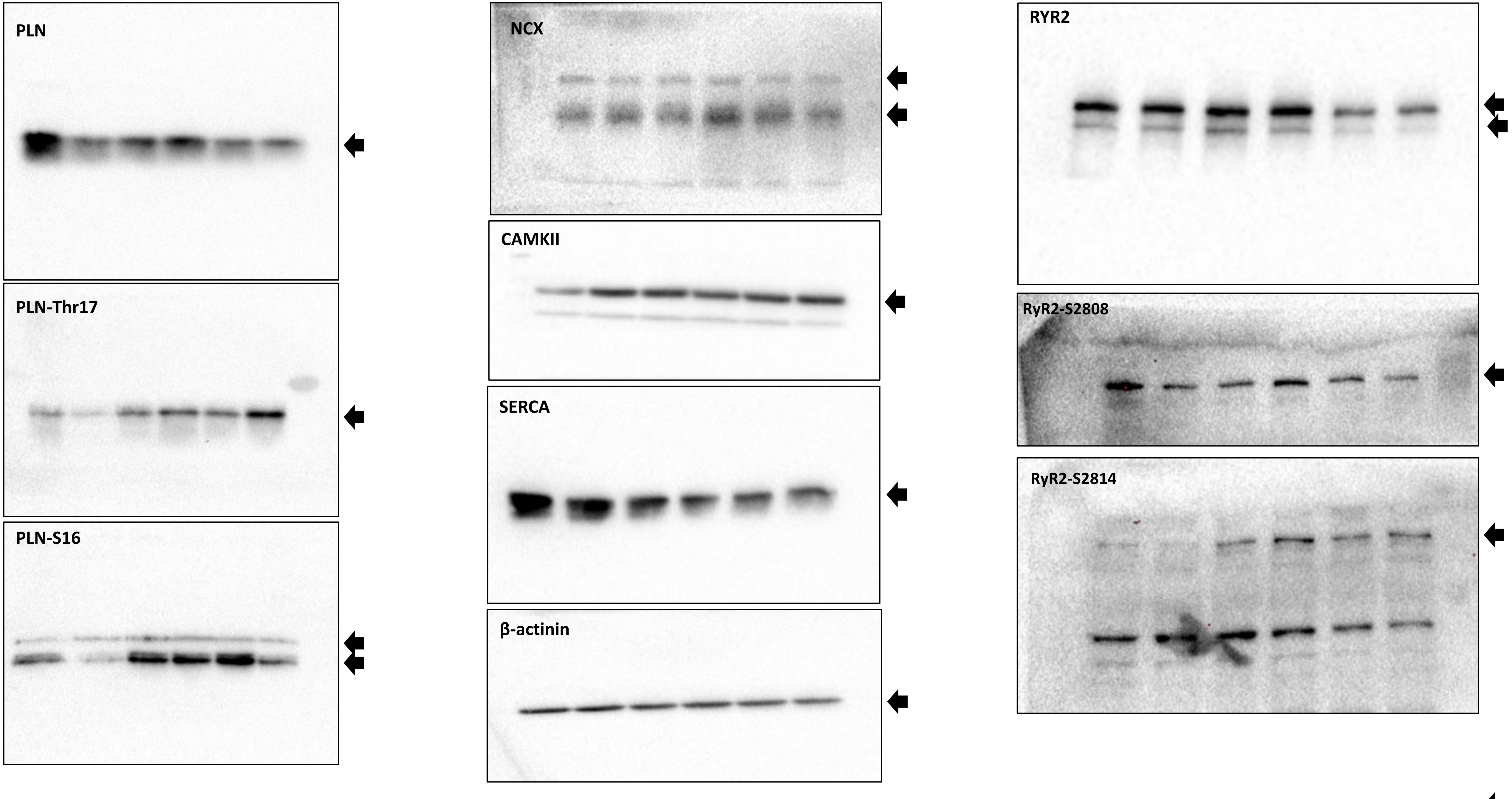

Control after DOX treatment, Fig. 7I

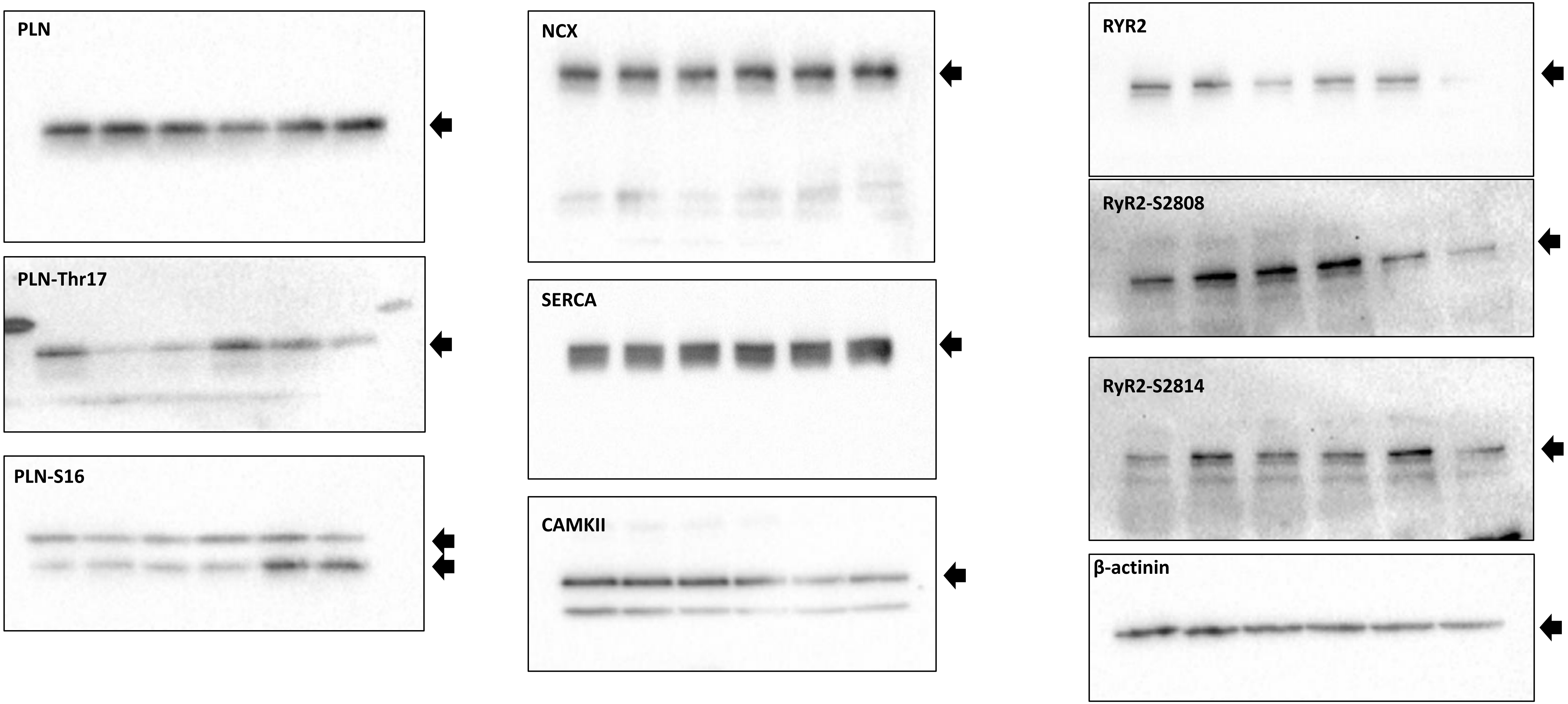

Basal expression of calcium handling proteins, Supplementary Figure 5B

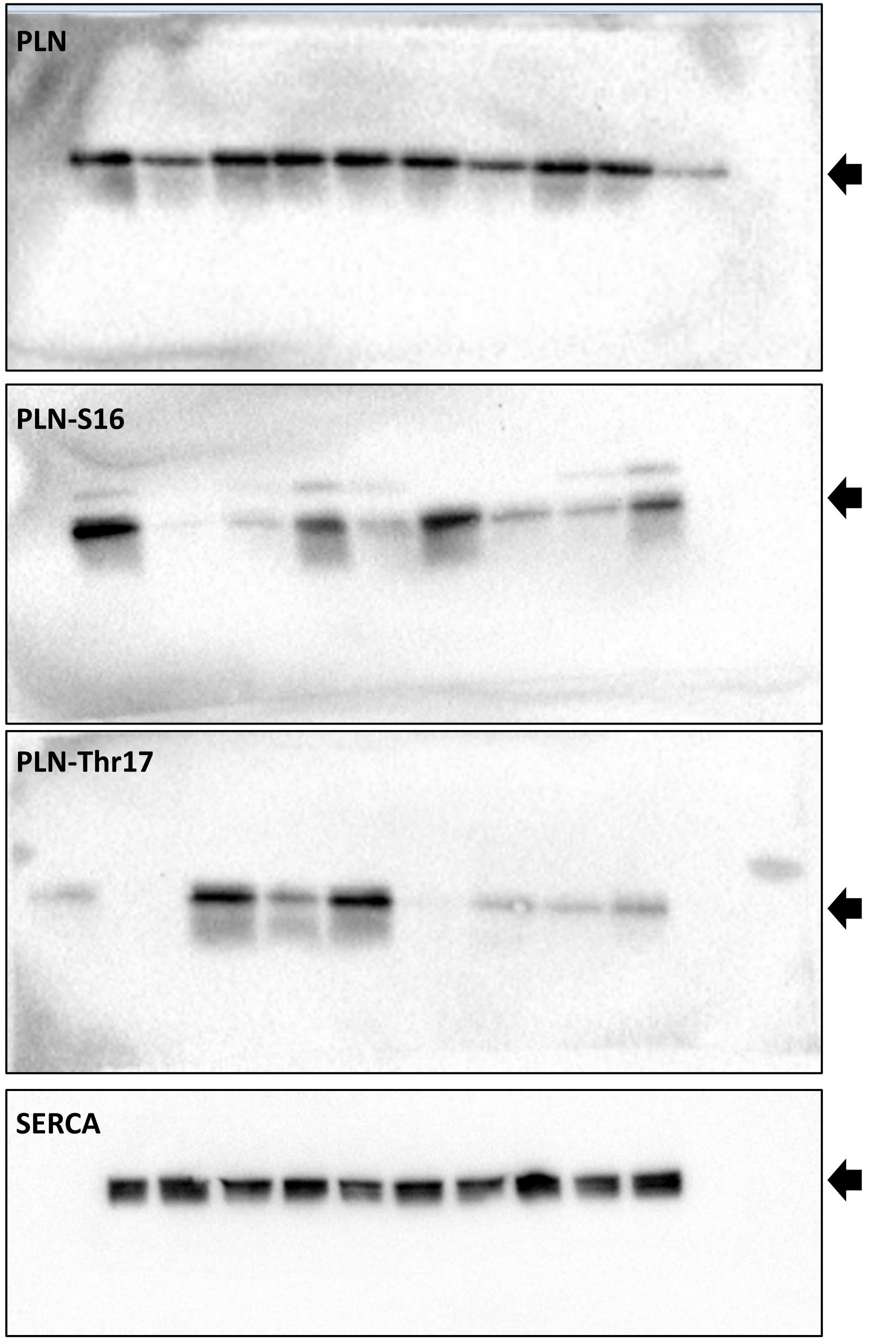

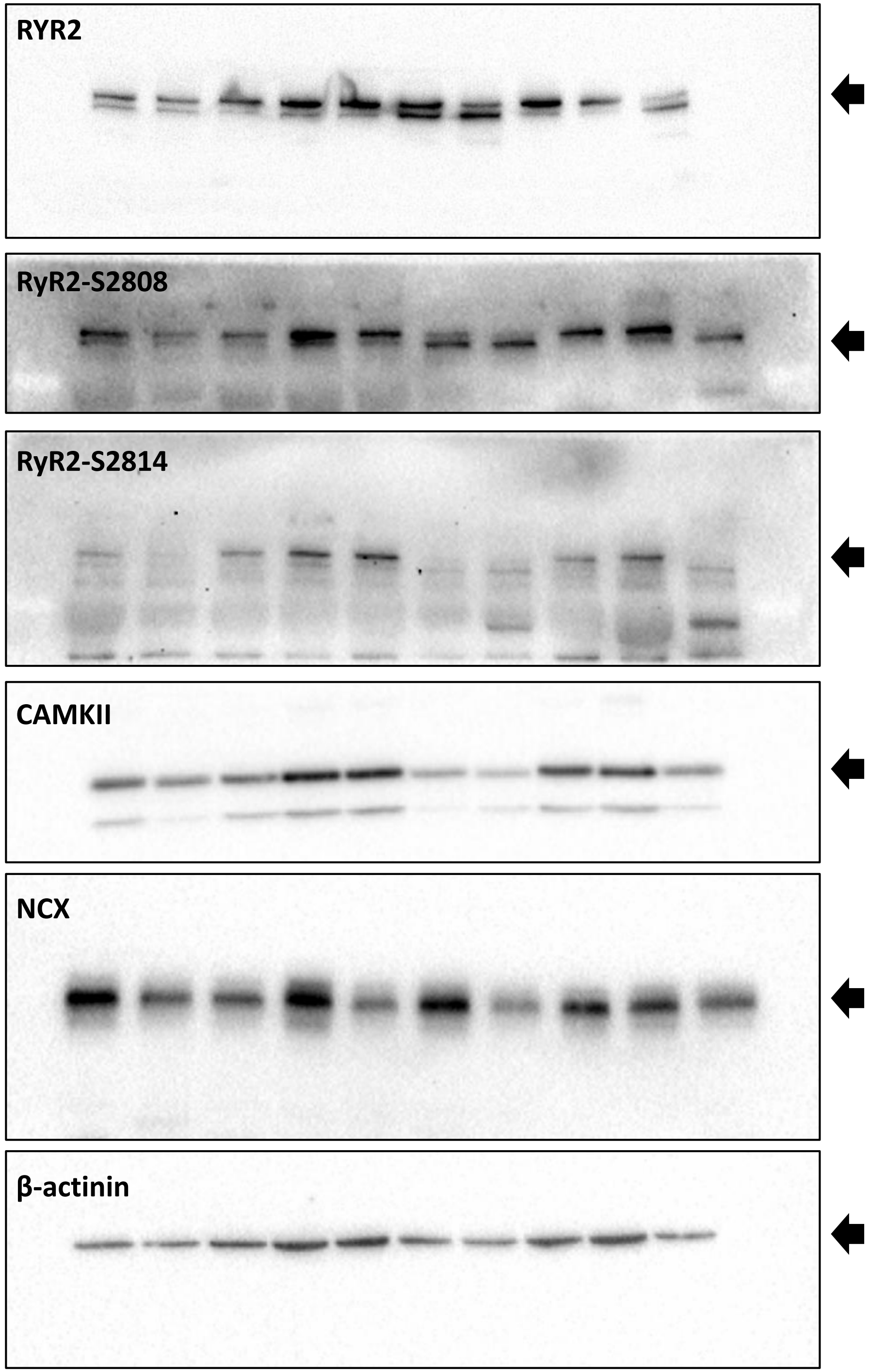

Supplement: Supplementary file 1 — Supplementary file1 (PDF 688 KB) [file 395_2022_918_MOESM1_ESM.pdf]
