# Supplementary material for: Doxorubicin induces cardiotoxicity in a pluripotent stem cell model of aggressive B cell lymphoma cancer patients
Source: Basic Res Cardiol. 2022 Mar 8;117(1):13. doi: 10.1007/s00395-022-00918-7 (PMC8904375; doi:10.1007/s00395-022-00918-7)
Supplement: Supplementary file 7 — Supplementary file7 (DOCX 74 KB) [file 395_2022_918_MOESM7_ESM.docx]

**Supplementary material of**

**Doxorubicin induces cardiotoxicity in a pluripotent stem cell model of aggressive B cell lymphoma cancer patients**

**Short title: Mechanisms of DOX-induced ACT**

Luis Peter Haupt^a#^; Sabine Rebs^a#^, Wiebke Maurer^a^, Daniela Hübscher^a^, Malte Tiburcy^c^; Steffen Pabel^d^, Andreas Maus^a,b^, Steffen Köhne^a^; Rewati Tappu^e^; Jan Haas^e^; Yun Li^f^; Andre Sasse^g^_;_ Celio C. X. Santos^b^; Ralf Dressel^g^; L. Wojnowski^h^; Gertrude Bunt^i^; Wiebke Moebius^j, k^, Ajay M. Shah^b^; Benjamin Meder^e^; Samuel Sossalla^a,d^; Bernd Wollnik^f^; Gerd Hasenfuss^a^; Katrin Streckfuss-Bömeke^a,l,§^

^a^ Clinic for Cardiology and Pneumonology, University Medical Center Göttingen, Göttingen, Germany.

^b^ King’s College London British Heart Foundation Centre of Excellence, London, UK, United Kingdom

^c^ Institute of Pharmacology and Toxicology, University Medical Center Göttingen, Germany

^d^ Department of Internal Medicine 2 - Cardiology, University Medical Center Regensburg, Germany

^e^ Department of Cardiology, University of Heidelberg, Germany

^f^ Institute of Human Genetics, University Hospital Center Göttingen, Göttingen, Germany;

^g^ Institute of Cellular and Molecular Immunology, University Medical Center Göttingen

^h^ University Medical Center Mainz, Department of Pharmacology, Mainz, Germany

^I^ Clinical Optical Microscopy, University Medical Center Göttingen, Göttingen, Germany

^j^ Department of Neurogenetics, Electron Microscopy Core Unit, Max Planck Institute of Experimental Medicine, Göttingen, Germany.

^k^ Cluster of Excellence "Multiscale Bioimaging: from Molecular Machines to Networks of Excitable Cells" (MBExC), University of Göttingen, Göttingen, Germany

^l^ Institute of Pharmacology and Toxicology, Würzburg University, Würzburg, Germany

DZHK (German Center for Cardiovascular Research), partner site Göttingen (LH, SR, DH, AM, MT, SK, RD, SS, GH, K.S.-B), Partner site Heidelberg (RT, JH, BM)

^#^ equal contribution

**Supplementary Figure 1:** Overview of the used material

**Supplementary Figure 2.** Analysis of human myocardium of ACT patients and isolated ACT cardiac fibroblasts. **(A)** qPCR analysis of non-failing (cFB-NF) and diseased cFB (cFB-ACT), patient sFB, iPS-cardiomyocytes from ACT patients (iPS-CM-ACT), and end-stage human heart failure myocardium of the left ventricle. *TUBB* was used as reference gene. Expression of general fibroblast markers *CTGF*, Collagen type 1 (*COL1A1*), *ACTA2*, Periostin (*POSTN*). Statistical analysis was performed using 1-way ANOVA. * p < 0.05. Bars indicate mean values ± SEM. **(B)** Expression of fibroblast marker in diseased (ACT) cFB, and sFB with antibodies against α-SMA (green) and COL1 (red). DAPI (blue) was used for nuclear staining. Scale bar: 50 μm.

**Supplementary Figure 3.** Generated iPSCs show stem cell characteristics, and express stem cell markers. **(A)** iPSC show characteristic stem cell morphology, stain positive for alkaline phosphatase (AP; red), and express the stem cell markers OCT4, SOX2, NANOG, LIN28, SSEA4, and TRA-1-60 (depicted images are representative for all cell lines). Nuclei were stained with DAPI (blue). Pictures of Control1 show stem cells on mouse embryonic fibroblasts (MEF). Control2 and Patients1-3 are depicted on feeder-free, Geltrex-coated surface. **(B)** Semiquantitative RT-PCR analysis showed mRNA expression of *NANOG, OCT4, LIN28, SOX2* and *GDF3* in all iPSC lines and in the iPSC positive control (FB1^1^). Low or no expression was observed in fibroblasts which were used for initial reprogramming. The housekeeping gene *GAPDH* served as reference, and H_2_O was used as negative control. **(C)** After spontaneous *in vitro* differentiation, iPSC-derived embryoid bodies express β-III-Tub (ectoderm), α-SMA (mesoderm) and AFP (endoderm) as shown by immunofluorescence staining. Nuclei were stained with DAPI (blue). Sections of embedded teratomas were stained with H&E and showed neuronal rosettes (ectoderm), muscle, connective or cartilage tissue (mesoderm), and intestinal or glandular tissue (endoderm). Scale bars: Brightfield and ALP: 200 μm; immunofluorescent stainings and H&E: 100 μm.

**Supplementary Figure 4:** **Characterization of iPSC CM show predominately ventricular cardiac differentiation.** **(A)** IPSC CM from control and ACT express cardiac markers α-actinin (green) and titin (red). **(B)** IPSC CM were stained with the cardiac marker cTNT and analyzed by FLOW cytometry. **(C)** qPCR analysis of IPSC CM for general cardiac markers *TNNT2, ACTN2, MYH6* *and MYH7. GAPDH* was used as a reference and undifferentiated iPSC serve as a negative control. **(D)** IPSC CM from control and ACT express the ventricular cardiac marker MLC2v (red), but lack atrial cardiac marker MLC2a (green). **(E)** Quantification of atrial vs ventricular iPSC CM using the immunofluorescence double-staining MLC2v/MLC2a (v-iPSC CM: 172 cells of 3 differentiations; a-iPSC CM: 206 cells of 3 differentiations). Statistical analysis by Two-Way ANOVA with Sidak’s multiple comparison. Representative atrial differentiated iPSC CM express the atrial cardiac marker MLC2a (green), but lack the ventricular cardiac marker MLC2v (red) (right). **(F)** qPCR analysis of iPSC CM from ACT (patient 1, 2, 3) and control (control 1, 2) for the ventricular marker *MLC2v*, the atrial markers *NR2F2* and *PITX2*. Independent control iPSC were differentiated in the ventricular as well as atrial directions and served as positive or negative control (PC; NC; v-iPSC CM; a-iPSC CM). iPSC from ACT patients also served as negative control. 2-3 different cardiac differentiations were analyzed per cell line. v: ventricular, a: atrial. Different colored dots indicate the different control CM (green: control 1, grey: control 2) and ACT CM (blue: ACT1, black: ACT2 and red: ACT3) used. Scale bar: 50 μm (A, B); 100 µM (E).

**Supplementary Figure 5: Proliferation in iPSC and iPSC CM with EdU incorporation assay.**

**(A)** Example of immunofluorescence images of iPSC and iPSC CM from ACT3. The iPSC are stained with DAPI (blue/nucleus), EdU (green/proliferating cells) and LIN28 (red/ pluripotency marker). The iPSC CM are stained with DAPI (blue/nucleus), EdU (green/proliferating cells) and MLC2v (red/cardiac marker). Scale bars: 100 µM. **(B)** Quantification of EdU positive cells. The iPSC show strong proliferation with 100 %, whereas the proliferation of iPSC CM is significantly decreased. Data is represented as scatter blot with n=10 images analyzed for each line. Statistical analysis was performed by Mann-Whitney test whereas the iPSC was compared against the respective iPSC CM cohort. Results are marked by * ***p<0.001.

**Supplementary Figure 6.** **DOX dependent alterations on a patient-specific level. (A-C)** Data is shown as scatter blots for every control and ACT CM separately. **(A)** Effect of DOX on sarcomeric regularity in iPSC CM. Quantification of DOX-treated (24 h) sarcomeric regularity using Fast Fourier Transform algorithm. **(B)** DOX-dependent cell viability of iPSC CM using the Casy@Roche-System. Quantification of cell viability in % in iPSC-CM. The cell viability is not influenced in iPSC-CM after treatment with 0.25 µM DOX for 24 h. Sample numbers: 4 control-iPSC CM batches from 1 differentiation, 12 patient iPSC CM batches from 3 differentiations. Different colored dots indicate the different control-lines (grey: control 2) and patient lines (blue: ACT1 and black: ACT2) used. Statistical analysis was performed by Mann-Whitney test. Bars indicate mean values with SEM. * p < 0.05. **(C)** Effect of DOX on iPSC CM cell death. Annexin V/PI apoptosis tests showed that the number of apoptotic and dead cells rose with increasing DOX levels. **(D)** The amount of H_2_O_2_ in the supernatant of iPSC-CM was measured with the Amplex Red assay after 24 h DOX treatment. Different colored dots indicate the different control-lines (green: control 1, grey: control 2) and patient lines (blue: ACT1, black: ACT2 and red: ACT3) used. For sample numbers see Supplementary Table 10. Statistical analysis was performed using 2-way ANOVA with Tukey’s multiple comparison (A, C) or multiple T-tests (D). Data are shown as mean + SEM. * p < 0.05, ** p < 0.01, *** p < 0.001, ****p<0.0001.

**Supplemental Figure 7.** **EHM from control and ACT CM show altered beating activity dynamics after DOX treatment. (A)** The percentage of control and ACT EHM that depict an irregular spontaneous beating activity is higher after DOX treatment. **(B)** The cross-sectional area of control EHMs is larger than the one of ACT EHM. **(B-E)** Analysis of physiological parameters in EHM. For control EHM, 6 independent EHM from 1 cell line of control 1 and 2, and for ACT patient EHM, 5 independent EHM from 1 cell line of ACT1 and 6 independent EHM of each ACT2 and ACT3. All measurements are shown separately for every control and ACT patient for beats per minute **(C)**, force of contraction **(D)** and normalized force of contraction **(E)**. Statistical analysis was performed using 2-way ANOVA (Data are shown as mean + SEM. * p < 0.05, ** p < 0.01, *** p < 0.001.

**Supplemental Figure 8.** **Basal protein expression of calcium handling genes.** (**A**) Basal protein expression of SERCA, NCX, CAMKIIδ, PLN, PLN-S16p, PLN-Thr17p and RYR2 in iPSC CM of ACT patients and controls. (**B**) Representative western blot results used for quantification of patient and control iPSC-CMs. 11 Ctrl-iPSC CM differentiations, 18 ACT patient-iPSC CM differentiations. Statistical analysis was performed using Students’ t-test (D). Data are shown as mean + SEM. * p < 0.05, ** p < 0.01, *** p < 0.001.

**Material and methods**

**Supplementary Table 6**: Cells/ Cell lines used in this study

| **Somatic cells** | **Cell source** |
| --- | --- |
| 1-F | Skin fibroblasts from patient 1 |
| 2-F | Skin fibroblasts from patient 2 |
| 3-F | Skin fibroblasts from patient 3 |
| 1-C-F | Skin fibroblasts from control 1 |
| 2-C-F | Skin fibroblasts from control 2 |
| ACT cFB | Cardiac FB isolated from left ventricle of ACT4 myocardium, 65-year-old-female |
| Control cFB- | Lonza, NHCF-V Human Cardiac Fibroblast- Ventricular CC2904, 52-year-old-male |
| **iPSCs** |  |
| 1-ACT-1 | iPS cell line 1 generated from fibroblasts 1-F of ACT patient 1 |
| 1-ACT-2 | iPS cell line 2 generated from fibroblasts 1-F of ACT patient 1 |
| 2-ACT-1 | iPS cell line 1 generated from fibroblasts 2-F of ACT patient 2 |
| 2-ACT-2 | iPS cell line 2 generated from fibroblasts 2-F of ACT patient 2 |
| 3-ACT-1 | iPS cell line 1 generated from fibroblasts 3-F of ACT patient 3 |
| 3-ACT-2 | iPS cell line 2 generated from fibroblasts 3-F of ACT patient 3 |
| 1-C-1 | iPS cell line 1 generated from fibroblast of healthy donor 1 |
| 1-C-2 | iPS cell line 2 generated from fibroblast of healthy donor 1 |
| 2-C-1 | iPS cell line 1 generated from fibroblast of healthy donor 2 |
| 2-C-2 | iPS cell line 2 generated from fibroblast of healthy donor 2 |

**Supplementary Table :7 Primers used in RT-PCR and qPCR**

| Name | Sequence (5’ → 3’) |  | Purpose | bp |
| --- | --- | --- | --- | --- |
| *ACTA2* | For: | AAGCACAGAGCAAAAGAGGAAT | qRT-PCR | 76 |
|  | Rev: | ATGTCGTCCCAGTTGGTGAT |  |  |
| *ACTN2* | For: | AGG AGG AAG AAT GGC CTG AT | qRT-PCR | 291 |
|  | Rev: | GAT GCA GTA CTG GGC CTG AT |  |  |
| *COL1A1* | For: | AGACAGTGATTGAATACAAAACCA | qRT-PCR | 130 |
|  | Rev: | GGAGTTTACAGGAAGCAGACA |  |  |
| *CTGF* | For: | TTGGCAGGCTGATTTCTAGG | qRT-PCR | 193 |
|  | Rev: | GGTGCAAACATGTAACTTTTGG |  |  |
| *GAPDH* | For: | GTCTCCTCTGACTTCAACAGCG | qRT-PCR | 110 |
|  | Rev: | ACCACCCTGTTGCTGTAGCCAA |  |  |
| *GDF3* | For: | TTCGCTTTCTCCCAGACCAAGGTTTC | PCR | 311 |
|  | Rev: | TACATCCAGCAGGTTGAAGTGAACAGCACC |  |  |
| *HPRT* | For: | CAAAGATGGTCAAGGTCGC | qRT-PCR | 81 |
|  | Rev: | CAAATCCAACAAAGTCTGGCT |  |  |
| *LIN28* | For: | AGTAAGCTGCACATGGAAGG | PCR | 410 |
|  | Rev: | ATTGTGGCTCAATTCTGTGC |  |  |
| *MLC2v* | For: | GGCGAGTGAACGTGAAAAAT | qRT-PCR | 200 |
|  | Rev: | CAGCATTTCCCGAACGTAAT |  |  |
| *MMP9* | For: | GCACGACGTCTTCCAGTACC | qRT-PCR | 124 |
|  | Rev: | CAGGATGTCATAGGTCACGTAGC |  |  |
| *MYH6* | For: | GTCATTGCTGAAACCGAGAATG | qRT-PCR | 413 |
|  | Rev: | GCAAAGTACTGGATGACACGCT |  |  |
| *MYH7* | For: | AGACTGTCGTGGGCTTGTATCAG | qRT-PCR | 101 |
|  | Rev: | GCCTTTGCCCTTCTCAATAGG |  |  |
| *NANOG* | For: | AGTCCCAAAGGCAAACAACCCACTTC | PCR | 164 |
|  | Rev: | ATCTGCTGGAGGCTGAGGTATTTCTGTCTC |  |  |
| *NCF4* | For: | CCTCCTCAGTCGGATCAACAA | qRT-PCR | 181 |
|  | Rev: | TGACGTCGTCTTTCCTGATGA |  |  |
| *NOX4* | For: | GCAGGAGAACCAGGAGATTG | qRT-PCR | 125 |
|  | Rev: | CACTGAGAAGTTGAGGGCATT |  |  |
| *NR2F2* | For: | CCGACCGGGTGGTCGCCTTTATGGA | qRT-PCR | 223 |
|  | Rev: | CGGCTGGTTGGGGTACTGGCTCCTA |  |  |
| *OCT4* | For: | GACAACAATGAAAATCTTCAGGAG A | PCR | 473 |
|  | Rev: | TTCTGGCGCTTACAGAACCA |  |  |
| *PITX2* | For: | AGCCATTCTTGCATAGCTCG | qRT-PCR | 108 |
|  | Rev: | GTGTGGACCAACCTTACGGA |  |  |
| *POSTN* | For: | ACAAGAAGAGGTCACCAAGGTC | qRT-PCR | 109 |
|  | Rev: | CTTCCTCACGGGTGTGTCTC |  |  |
| *RAC2* | For: | AAGAAGCTGGCTCCCATCACCTAC | qRT-PCR | 113 |
|  | Rev: | AACACGGTTTTCAGGCCTCTCTG |  |  |
| *SERCA2a* | For: | ACAGAGTGGAAGGTGATACTTGTTC | qRT-PCR | 384 |
|  | Rev: | AGTAAACCGACATTGACTTTCTGTC |  |  |
| *SOX2* | For: | ATGCACCGCTACGACGTGA | PCR | 437 |
|  | Rev: | CTTTTGCACCCCTCCCATTT |  |  |
| *TNNT2* | For:  Rev: | GAC AGA GCG GAA AAG TGG GA  TGA AGG AGG CCA GGC TCT AT | PCR, qRT-PCR | 305 |

bp: Base pairs

### **Supplementary Table 8: Antibodies used in Immunocytochemistry and Western experiments**

| Antigen | Host and isotype | Dilution |  | Supplier |
| --- | --- | --- | --- | --- |
|  |  | **WB** | **IF** |  |
| AFP | Rabbit, IgG | - | 1:100 | Dako A0008-4oC |
| CamKII | Rabbit, IgG | 1:5000 | - | Thermo fisher scientific PA5-22168 |
| cTNT | Mouse, IgG | - | 1:500 | Thermo Fisher Scientific MS295PABX 13-11 |
| GAPDH | Mouse, IgG | 1:500 | - | Millipore MAB374 |
| LIN28 | Goat, IgG | - | 1:300 | R&D systems AF3757 |
| MLC2a | Mouse, IgG | - | 1:200 | Synaptic Systems 311 011 |
| MLC2v | Rabbit, IgG | - | 1:200 | Proteintech 10906-1-AP |
| NANOG | Goat, IgG | - | 1:200 | Abcam PA5-18406 |
| NCX | Rabbit, IgG | 1:5000 | - | Swant ∏ 11-13 |
| OCT4 | Goat, IgG | - | 1:40 | R&D systems AF1759 |
| PLN | Mouse, IgG | 1:5000 | - | Thermo fisher scientific MA3-922 |
| PLN Ser16p | Rabbit, IgG | 1: 5000 | - | Badrilla A010-12AP |
| PLN Thr17p | Rabbit, IgG | 1:5000 | - | Badrilla A010-13 |
| RYR2 | Rabbit, IgG | 1:5000 | - | Sigma-Aldrich HPA020028 |
| RYR2 Ser2808p | Rabbit, IgG | 1:1000 | - | Badrilla A010-30 |
| RYR2 Ser2814p | Rabbit, IgG | 1:5000 | - | Badrilla A010-31 |
| SERCA | Mouse, IgG | 1:20,000 | - | Thermo fisher scientific MA3-919 |
| SOX2 | Mouse, IgG | - | 1:50 | R&D systems MAB2018 |
| SSEA4 | Mouse, IgG | - | 1:200 | Abcam MC813 |
| TRA-1-60 | Mouse, IgM | - | 1:200 | R&D systems MAB4770 |
| Titin-M8/M9 | Rabbit, IgG | - | 1:750 | Myomedix (order form) |
| α-actinin | Mouse, IgG | 1:10,000 | 1:1000 | Sigma-Aldrich A7811 |
| α-SMA | Mouse, IgG | - | 1:3000 | Sigma-Aldrich A2547 |
| β-actin | Mouse, IgG | 1:5000 | - | Santa Cruz sc-47778 |
| β-III-Tub | Mouse, IgG | - | 1:2000 | Covance MMS-435P |

### Secondary antibodies and fluorophore-conjugated probes

| Antigen | Conjugate | Host | Dilution | Supplier |
| --- | --- | --- | --- | --- |
| Mouse IgM | FITC | Goat | 1:200 | Jackson ImmunoResearch  Laboratories 115-095-020 |
| Rabbit IgG | Cy3 | Goat | 1:600 | Jackson ImmunoResearch  Laboratories 111-165-045 |
| Goat IgG | Alexa Fluor 555 | Donkey | 1:1000 | Thermo Fisher Scientific A21432 |
| Mouse IgG | Alexa Fluor 488 | Donkey | 1:1000 | Thermo Fisher Scientific A21202 |
| Mouse IgG | HRP | Donkey | 1:10.000 | Thermo Fisher Scientific A16011 |
| Rabbit IgG | HRP | Donkey | 1:10.000 | Thermo Fisher Scientific A16023 |
|  |  |  |  |  |

**Supplementary Table 9: Genetic variants found in ACT patients 1-3 by using whole exome sequencing**

| **Gene** | **Patients with ACT development** | | |
| --- | --- | --- | --- |
|  | **ACT1** | **ACT2** | **ACT3** |
| Cardiomyopathy-associated genes | | | |
| *PRDM16* |  | p.P878L(y) |  |
| *TTN* |  |  | p.V27642M |
| Neuromuscular disease- associated gene | | | |
| *SYNM* |  |  | p.V1004I |
| Cancer-associated gene | | | |
| *EGFR* |  |  | p.E868Nfs*35 |

**Supplementary methods**

**Spontaneous iPSC-differentiation into three germ layers:** Spontaneous differentiation into different germ layers shows the pluripotent capacity of iPSC. Our media did not contain factors that direct the differentiation into a specific cell-type. To generate embryoid bodies (EBs), iPSC of about 80% confluence (grown on mouse embryonic fibroblasts (MEF) feeder layer) were transferred to a non-coated 6 cm suspension culture dish in DMEM/F12 with GlutaMAX with 20% Knock-Out Serum Replacement, 1 % non-essential amino acids (NEAA, Thermo Fisher Scientific), 1% β-Mercaptoethanol (Serva Electrophoresis) and 10 ng/mL recombinant human basic fibroblast growth factor (PeproTech), and then incubated at 37°C overnight. When EBs had formed, they were carefully washed with Iscove´s modified Dulbecco´s medium with GlutaMax (Thermo Fisher Scientific), transferred into a non-coated 6 cm suspension culture dish, and cultivated in Iscove medium with 20 % FBS, 1x NEAA, and 450 μM monothioglycerol (Sigma-Aldrich) in suspension at 37 °C for 8 days. The medium was changed on day 4 and 7 after the start of differentiation. On day 8 about 30 EBs were plated on 0.1 % gelatin-coated culture plates and on 0.1 % gelatin-coated culture plates containing glass coverslips, respectively. EBs were cultivated for another 25 days in Iscove medium, which was replaced every two days. EBs in the culture plates were used for semiquantitative RT-PCR; EBs on cover slips were used for immunofluorescence staining.

Teratoma generation and analysis: For *in vivo* differentiation, iPSC colonies were subcutaneously injected into eight weeks-old immune-deficient SCID mice, as described previously^2^. Teratoma formation was observed six to eight weeks after injection and confirmed with hematoxylin and eosin staining of teratoma slices.

**RNA isolation:** Pellets of iPSC, CM, embryonic bodies and human cardiac tissue, respectively, were snap frozen and stored at -80°C. The RNA of iPSC and embryoid bodies was isolated with the SV Total RNA Isolation System (Promega) according to the manufacturer’s protocol. In short, the pellets were lysed with 400-600 μl RNA lysis buffer supplemented with β-ME (200 μL/10 mL). Equal volumes of 95%-ethanol were added to the lysate and it was then transferred to a spin basket tube. The samples were washed with RNA wash solution and incubated with DNase I mix for 15 minutes at room temperature (RT). The reaction was stopped with 200 μL of DNase stop solution. The samples were washed twice and the RNA was eluted with 100 μL of nuclease free water. RNA concentration was determined with a spectrophotometer at 260/280 nm and the samples were stored at -80°C. For iPSC CM and human cardiac tissue the ReliaPrep RNA Tissue Miniprep System (Promega) was used as stated by the manufacturer’s protocol. For RNA isolation from human cardiac tissue, a piece of tissue approximately 4 mm³ in size and weighing approximately 8 - 15 mg was cut off with a scalpel in a bath of liquid nitrogen that had been filled into an aluminum bowl beforehand to avoid thawing. The piece was then transferred into a 1.5 ml reaction tube containing 500 µl of RNA lysis buffer and homogenized using the MICCRA D9-Homogenizer. Three steps of homogenization were performed, each for 8 seconds, with cooling on ice for 1 min between each step. RNA was eluted in a volume varying between 15 µl and 30 µl.

**Gene expression analysis**: MuLV reverse transcriptase (Thermo Fisher Scientific) was used according to the manufacturer's protocol to receive cDNA from our isolated RNA. Gene expression of pluripotency markers, cardiac markers, and germ layer markers was tested with semi-quantitative polymerase chain reaction (PCR) as described previously by our lab^3^. Detailed primer sequences, and reaction mixes for reverse transcription and PCR are listed in Supplementary Table 7.

Quantitative real-time PCR (qRT-PCR) was carried out as described previously^3^. In brief, DNA standards with concentrations ranging between 1 ng/μL to 0.125 fg/μL served as a reference. For our assays we used the 7900HT Fast Real-Time PCR System (Applied Biosystems). The PCR reaction went through 40 cycles of 15 sec at 95°C followed by 1 min of 60°C. Fluorescence was measured after each cycle. Specificity of the products was ensured through a melt curve analysis at the end of the run. Threshold cycles calculations and quantification of DNA was done by the system’s software iQ5.

**Protein isolation:** For Western blot analysis, CM pellets were lysed with 80-120 µl protein lysis buffer (12.5 µl 2 mol/L Tris-HCl (pH 7.4), 62.5 µl 4 mol/L NaCl, 125 µl 200 mmol/L NaF, 62.5 µl 20% Triton, 62.5 µl 20% IGEPAL® CA-630, 12.5 µl 1mmol/L Na_3_VO_4_, 12.5 µl 1mmol/L DTT, 125 µl PhosSTOP, 125 µl cOmplete EDTA-free, 650 µl dH_2_O). After an incubation time of 10 min on ice, the samples were vortexed and then centrifuged for 5 min at 5000 rpm and 4°C. The protein content of the supernatant was determined with the BCA Protein Assay Kit (Pierce) according to the manufacturer's recommendation. In short, 25 µl of diluted protein samples and the provided standard were pipetted in triplicates onto a 96-well plate. 200 μL of the BCA working solution were added and the samples were incubated for 30 min at 37°C. Afterwards, the absorption was detected photospectroscopically with a Mithras LB 940 plate reader (Berthold Technologies) at 562 nm. Protein samples were stored at -80°C.

**Western Blot:** Before loading, samples were heated to 37°C for 5 min. 15 μL lysate with 2 μg/μL protein in 1x SDS sample buffer were loaded into each well of a 15/8 % SDS–PAGE. The gel was run at a current of 30 mA. Proteins were transferred on a PVDF membrane with the semi-dry Trans-Blot Turbo Transfer System (Bio-Rad). Membranes were blocked with 5 % milk for 1 hour at room temperature and incubated with respective primary antibodies overnight at 4°C. Chemiluminescent HPR substrate was used to visualize antibody-labeled proteins. A detailed list of applied antibodies is provided in Supplementary Table 8. The intensity of individual bands from western blots was quantified using ImageJ software, normalized to GAPDH or α-actinin.

**Immunocytochemistry staining:** Occurrence and location of cellular proteins was shown with immunocytochemistry staining as described earlier by our group^3^. In short, iPSC or iPSC-CM were cultured on glass cover slips, fixed with 4 % paraformaldehyde (PFA, Sigma) in PBS at room temperature for 20 min and blocked with 1 % BSA/PBS overnight at 4°C. For stainings of intracellular proteins, cells were permeabilized with 0.1% Triton X-100 for 10 min at room temperature. All samples were incubated with the primary antibody overnight at 4 °C in a humidified chamber. For the secondary antibody the incubation time was 1 hour at 37 °C. The nuclei were counterstained with 4.6-diamino-2-phenylindole (DAPI, 0.2 ng/mL, Sigma) for 10 min at room temperature in the dark. Finally, the cover slips were mounted onto microscopy slides with Vectashield Mounting Medium (Liniaris Biologische Produkte GmbH) and sealed with nail polish. Images were captured with a Zeiss Axio Observer.Z1 microscope and a Zeiss AxioCam MRm 1.4MP camera. A detailed list of the used antibodies is attached (Supplementary Table 8).

**EdU incorporation assay:** EdU is a thymidine analog with an auxiliary ethinyl group used to allow monitoring of DNA synthesis and therefore proliferation. For this, 5 µM EdU was added to iPSC (at 30-40 % confluency) or to iPSC-CM (10^6^ cells) in a 12-well format. After 24 h, the cells were fixated and blocked for 1 h in a blocking buffer (10 % FCS, 1 % BSA and 0.3 % Triton-X in PBS) and afterwards the MLC2v antibody was added 1:200 in dilution buffer (1 % BSA and 0.3 % Triton-X in PBS) and incubated overnight at 4 °C. For visualization of the EdU incorporation, the Click-IT ™ EdU Cell Proliferation Kit (Thermo Fisher) was used. The primary antibody was discarded and 100 µl of Click-IT EdU mix containing 86 µl component D, 2 µl component E, 10 µl component F and 0.24 µl component B was added and incubated at room temperature for 30 min. Next, the secondary antibody was added (1:500 in dilution buffer) and incubated 1 h at 37 °C. Subsequently, the cells were mounted and imaged. For quantification, the number of EdU positive cells was divided by the total cell number in a picture and shown as percentage [%] of EdU positive cells.

**Apoptosis assay with flow cytometry:** The rate of apoptosis in our samples was observed with the APC-Annexin V Apoptosis Detection Kit with PI (BioLegend). 1.5 x 10^5^ iPSC CM were prepared according to the manufacturer's protocol. Briefly: For each condition we used 1.5 x 10^5^ iPSC-CMs. Cells were washed with PBS and incubated with 0.25 % Trypsin/EDTA for 5 min*.* To obtain a complete picture of apoptosis and necrosis, the supernatant was also transferred into a conical tube prepared with 500 μl FCS*.* The cells were segregated from the supernatant through centrifugation at 200 x g for 3 min. The samples were resuspended in 1 mL PBS and subsequently washed three times. After the last washing step, the cells were resuspended in Annexin V binding buffer. 100 μl of the suspension were transferred into each of four flow cytometry tubes. APC-Annexin V (5 μL) and propidium iodide (PI; 10 μL) were added to the tubes according to the following scheme: control (none), Annexin single staining (Annexin V), PI single staining (PI), and double staining (Annexin + PI). The mixes were incubated for 15 min in the dark at room temperature. Afterwards, 400 μl Annexin V binding buffer were added to each tube and the samples were measured with a BD FACSCanto II (BD Biosciences) flow cytometer. Unstained and single stainings served as references for Annexin-/PI-positive cell populations.

**Cell viability:** Cell viability of iPSC-CM was analyzed using a CASY cell counter (Roche). Therefore, 60 d old iPSC CM were digested onto wells of a 12 well plate with 40.000 CM per well. After 7 d, cells were treated with 0.25 µM DOX for 24 h and subsequently analyzed. For analysis, the CM were trypsinized, suspended in CASYon buffer and measured by the CASY system. The underlying principle of CASY is to measure and count the cells by applying a low-voltage field. Cell viability is determined by integrity of the plasma membrane. The low-voltage applied to the cells can’t pass through intact cells with an intact plasma membrane, therefore identifying them as a “viable” cell and vice versa.

**Cellular ROS generation:** Extracellular H_2_O_2_ in the supernatant of iPSC CM was assessed with an Amplex Red Hydrogen Peroxide/Peroxidase Assay Kit (Thermo Fisher Scientific). Fluorescence was measured at 540 nm excitation and 620 nm emission with a Berthold LB 940 Mithras plate reader.

**RNA sequencing (RNA-seq) and bioinformatics:** Diﬀerential gene expression was obtained by use of RNA sequencing performed on an Illumina HighSeq-2000 platform and bioinformatics. Total RNA was isolated from 2-month-old 3D EHM from 2 controls with 2-3 EHM each (n = 5-6 samples) and from 3 ACT-EHM (n = 6-7 samples) using standard protocols (Promega). 150 ng RNA per sample was subjected to library preparation (TruSeq RNA Library Prep Kit v2, Illumina) and next-generation RNA sequencing using paired-end sequencing (2 × 75 bp, > 40 Mio reads/sample) by the application of Illumina HiSeq2000 platform. The quality was checked with the tool FastQC [https://www.bioinformatics.babraham.ac.uk/projects/fastqc/]. The analysis of RNA-seq data was performed as described previously^4^. In brief, RNA-seq raw reads were aligned to the human grch38 genome assembly using the hisat2 tool with the option to output spliced alignments^5^. The SAM files thus obtained were converted to BAM files, sorted and indexed using Samtools ^6^. FeatureCounts program ^7^ with the paired-end option was used for counting the reads overlapping the features as described in the human GTF file (Homo_sapiens.GRCh38.90.gtf) obtained from Ensemble. The read counts table was imported in R and the DEseq2 package was used for reporting the normalized and variance stabilised counts as well as for performing the differential gene-expression analysis^8^. Differential expression analysis was performed for: a. DOX-treated vs. untreated controls; b. DOX-treated vs. untreated patients; c. DOX-treated patients vs. DOX-treated controls. Genes with a Benjamin and Hochberg–adjusted P value (false discovery rate) of < 0.05 were considered to be diﬀerentially expressed. Gene ontology (GO) enrichment analysis of bioprocesses was performed with GOSeq (FDR p-adjusted value < 0.05) <https://bioconductor.org/packages/release/bioc/vignettes/goseq/inst/doc/goseq.pdf>) )^9^.

**References**

1. Streckfuss-Bomeke K, Wolf F, Azizian A, Stauske M, Tiburcy M, Wagner S, et al. Comparative study of human-induced pluripotent stem cells derived from bone marrow cells, hair keratinocytes, and skin fibroblasts. *European heart journal*. 2013;34:2618-2629

2. Guan K, Nayernia K, Maier LS, Wagner S, Dressel R, Lee JH, et al. Pluripotency of spermatogonial stem cells from adult mouse testis. *Nature*. 2006;440:1199-1203

3. Borchert T, Hubscher D, Guessoum CI, Lam TD, Ghadri JR, Schellinger IN, et al. Catecholamine-dependent beta-adrenergic signaling in a pluripotent stem cell model of takotsubo cardiomyopathy. *Journal of the American College of Cardiology*. 2017;70:975-991

4. Halder R, Hennion M, Vidal RO, Shomroni O, Rahman RU, Rajput A, et al. DNA methylation changes in plasticity genes accompany the formation and maintenance of memory. *Nat Neurosci*. 2016;19:102-110

5. Kim D, Langmead B, Salzberg SL. Hisat: A fast spliced aligner with low memory requirements. *Nat Methods*. 2015;12:357-360

6. Li H, Handsaker B, Wysoker A, Fennell T, Ruan J, Homer N, et al. The sequence alignment/map format and samtools. *Bioinformatics*. 2009;25:2078-2079

7. Liao Y, Smyth GK, Shi W. Featurecounts: An efficient general purpose program for assigning sequence reads to genomic features. *Bioinformatics*. 2014;30:923-930

8. Love MI, Huber W, Anders S. Moderated estimation of fold change and dispersion for rna-seq data with deseq2. *Genome Biol*. 2014;15:550

9. Young MD, Wakefield MJ, Smyth GK, Oshlack A. Gene ontology analysis for rna-seq: Accounting for selection bias. *Genome Biol*. 2010;11:R14
