# Supplementary material for: Doxorubicin induces cardiotoxicity in a pluripotent stem cell model of aggressive B cell lymphoma cancer patients
Source: Basic Res Cardiol. 2022 Mar 8;117(1):13. doi: 10.1007/s00395-022-00918-7 (PMC8904375; doi:10.1007/s00395-022-00918-7)

Supplementary Figure 1: Overview of the used material

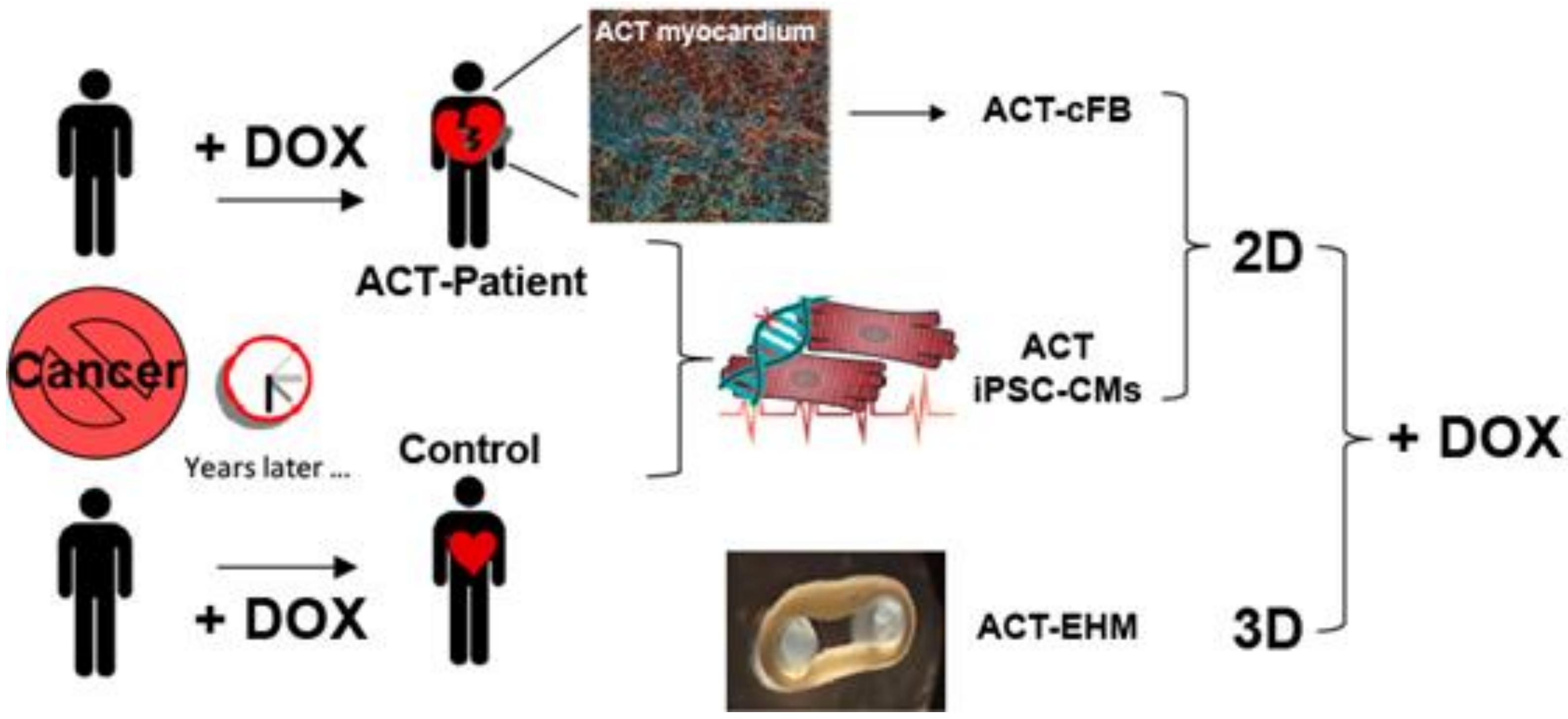

Supplementary Figure 2: Human ACT myocardium_cFB

A: Human isolated cardiac fibroblasts from ACT myocardium

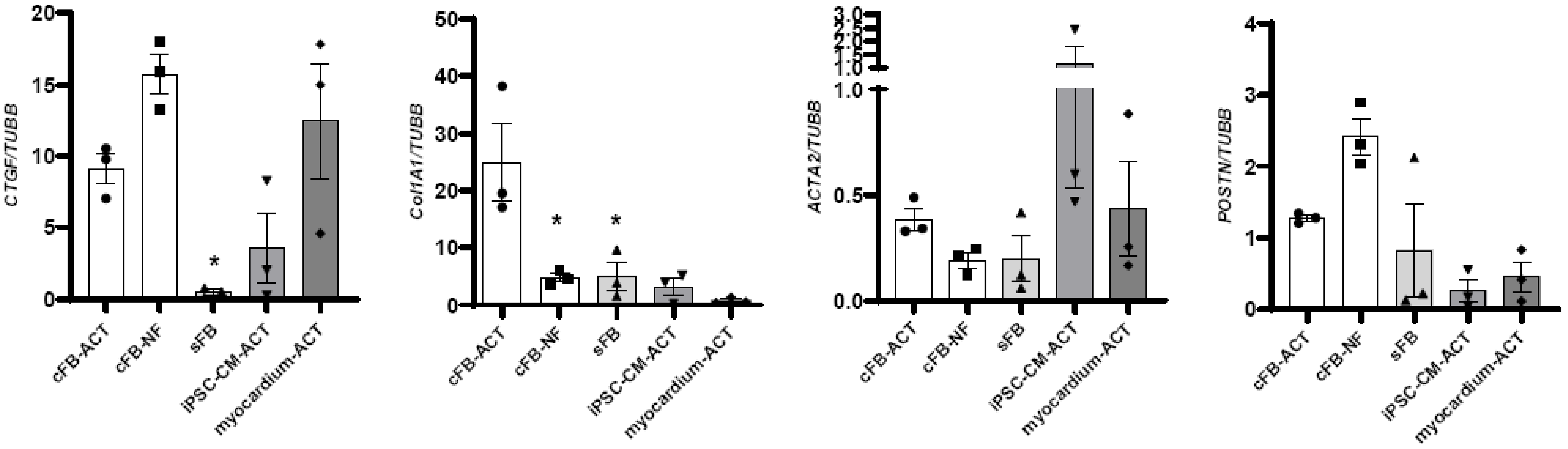

B: ACT-cFB

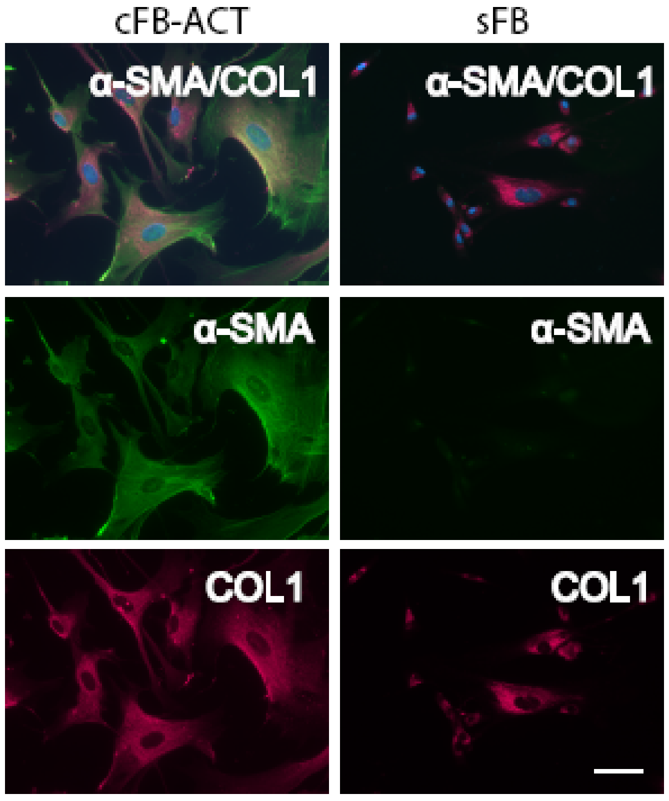

Supplementary Figure 3: Characterization of generated iPSC

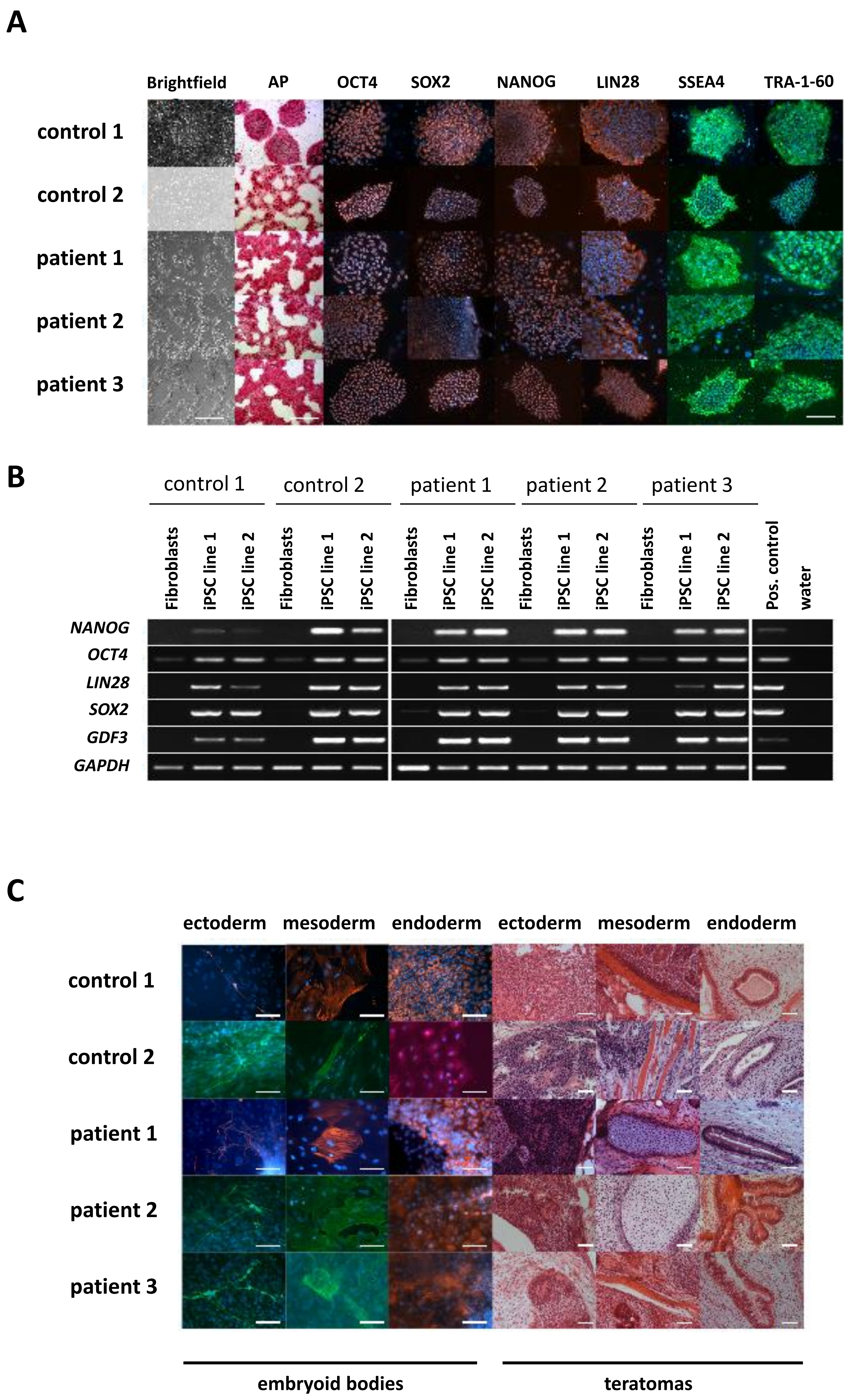

Supplementary Figure 4: Characterization of generated ventricular iPSC-CM

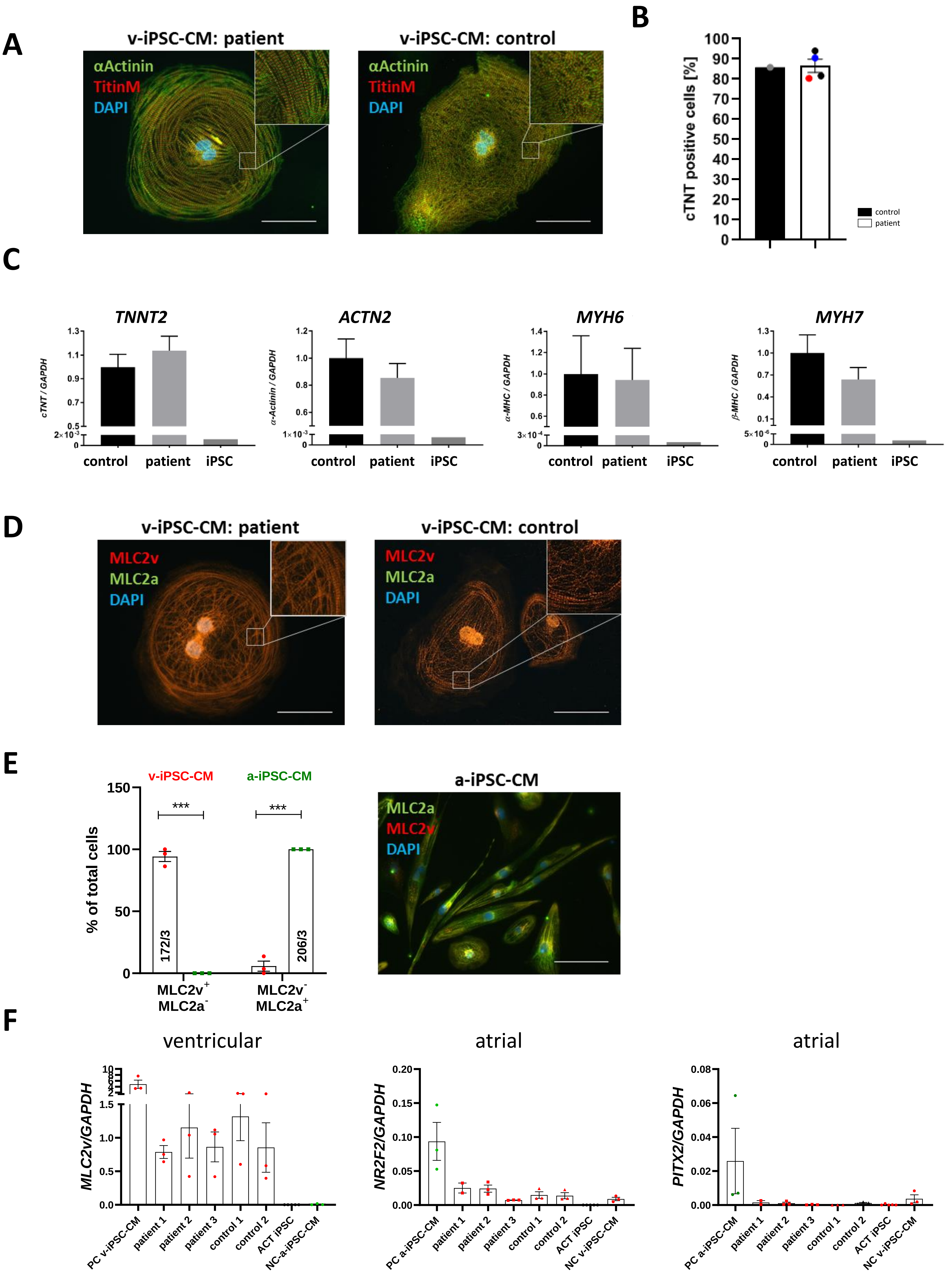

Supplementary Figure 5: Cell arrest in iPSC-CM and iPSC

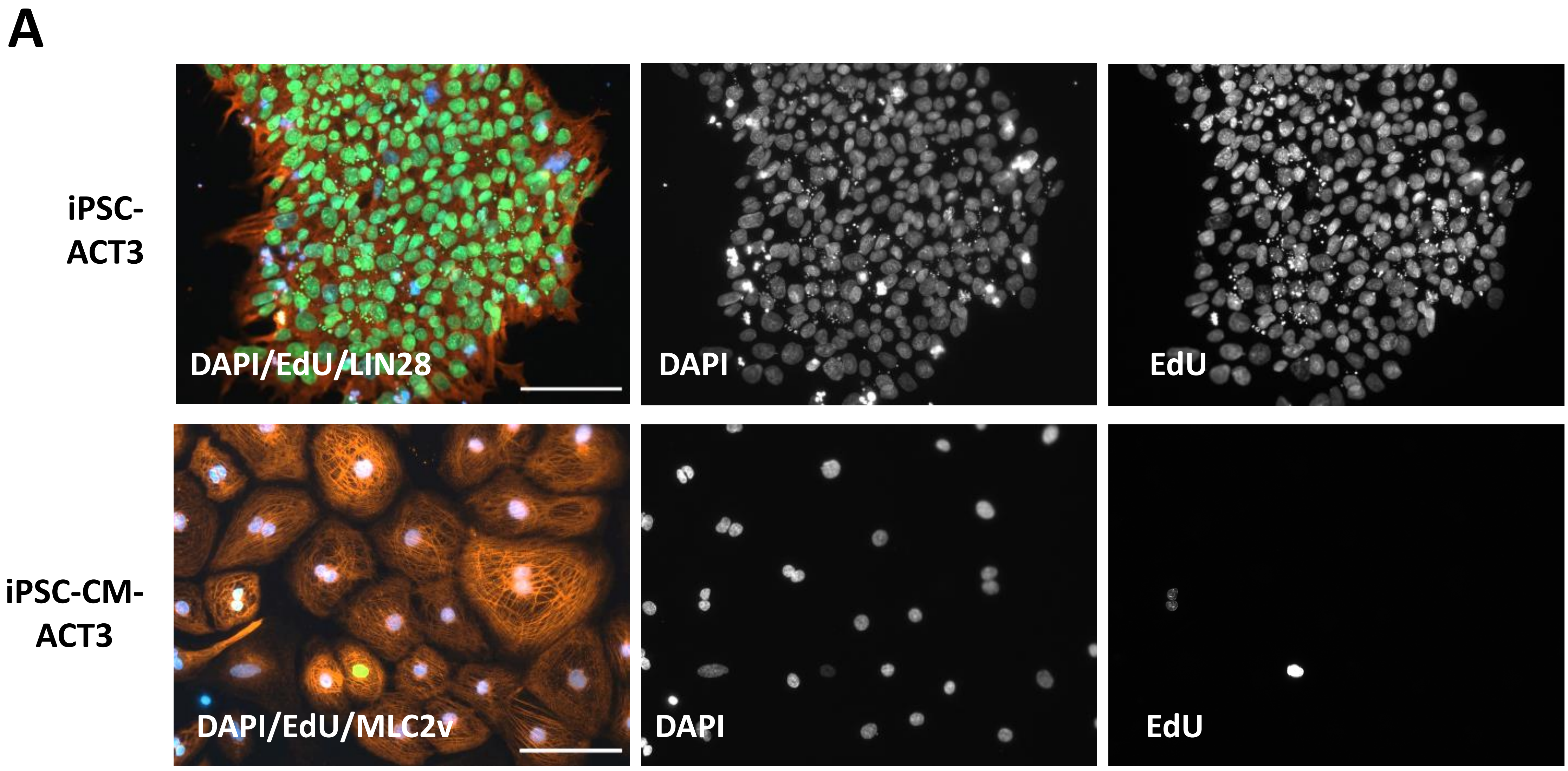

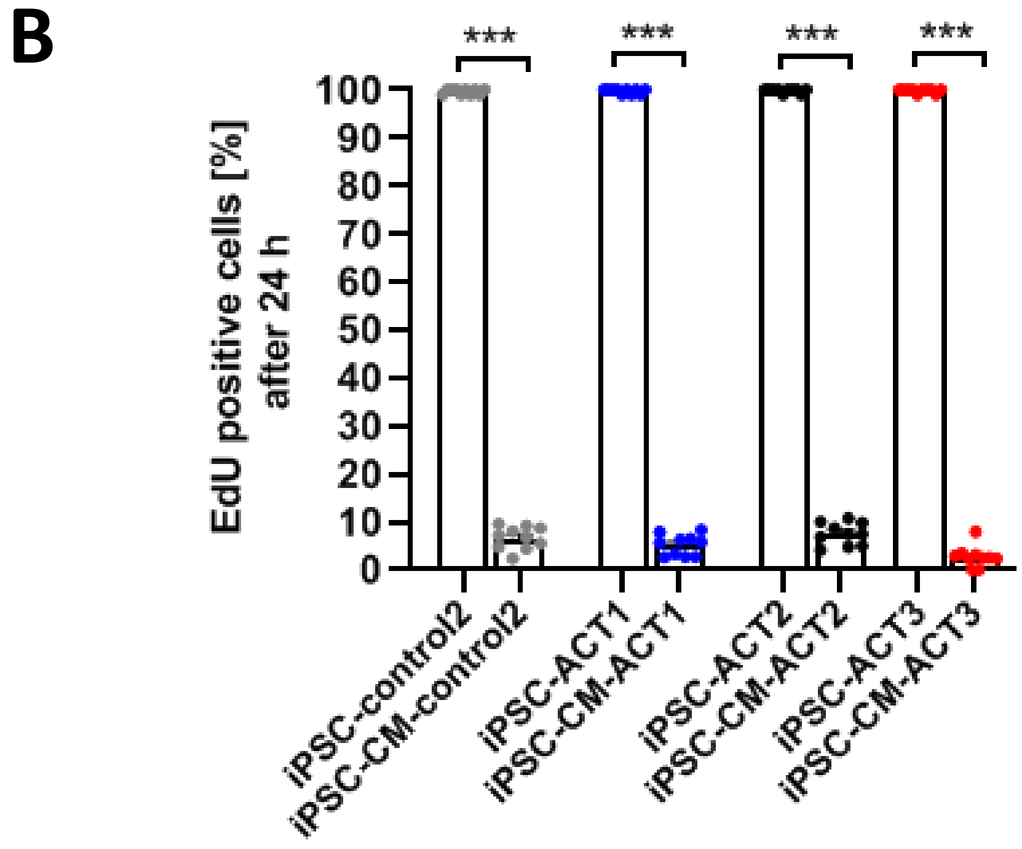

Supplementary Figure 6: DOX-dependent alterations on a patient-specific level

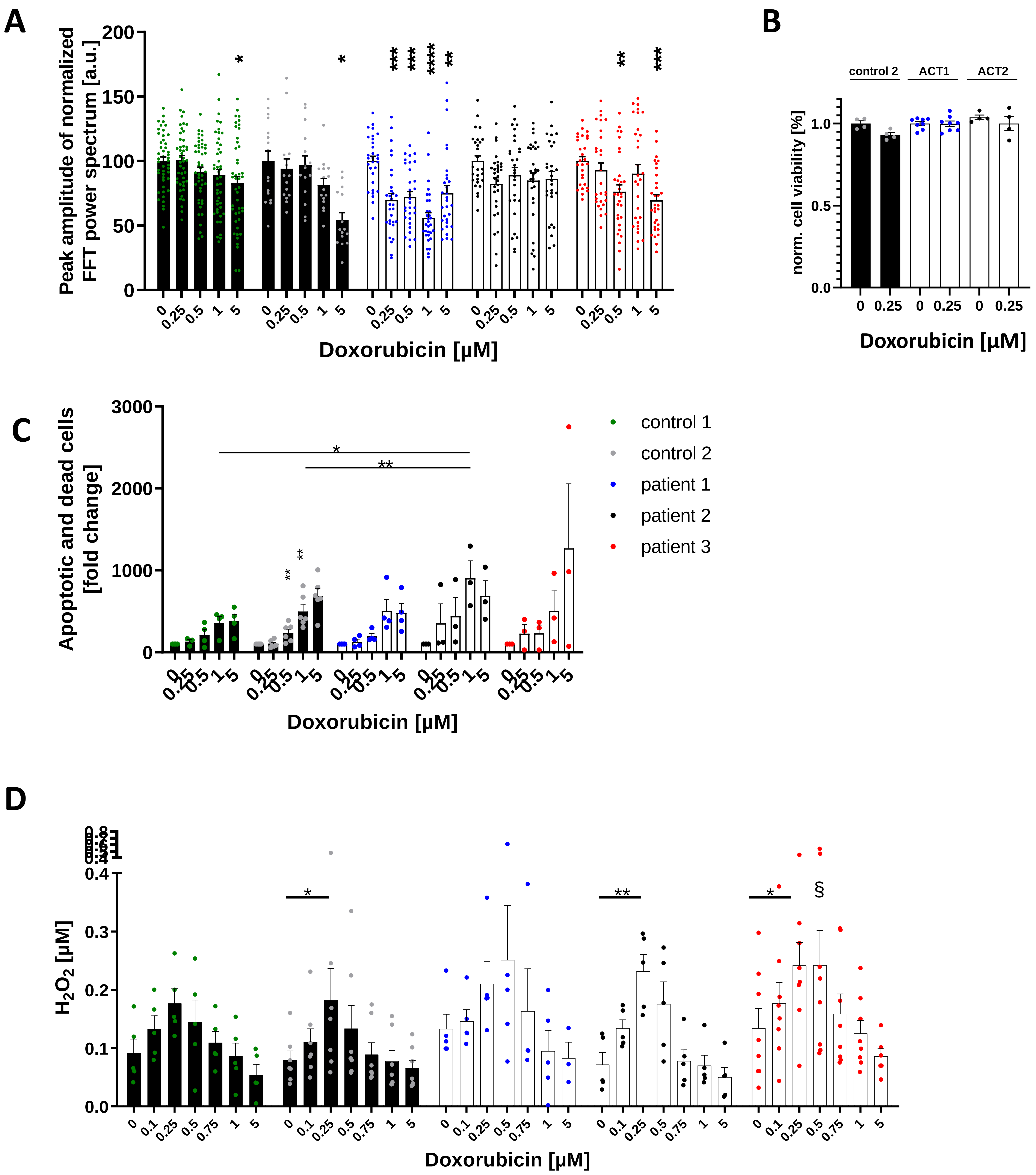

Supplementary Figure 7: EHM

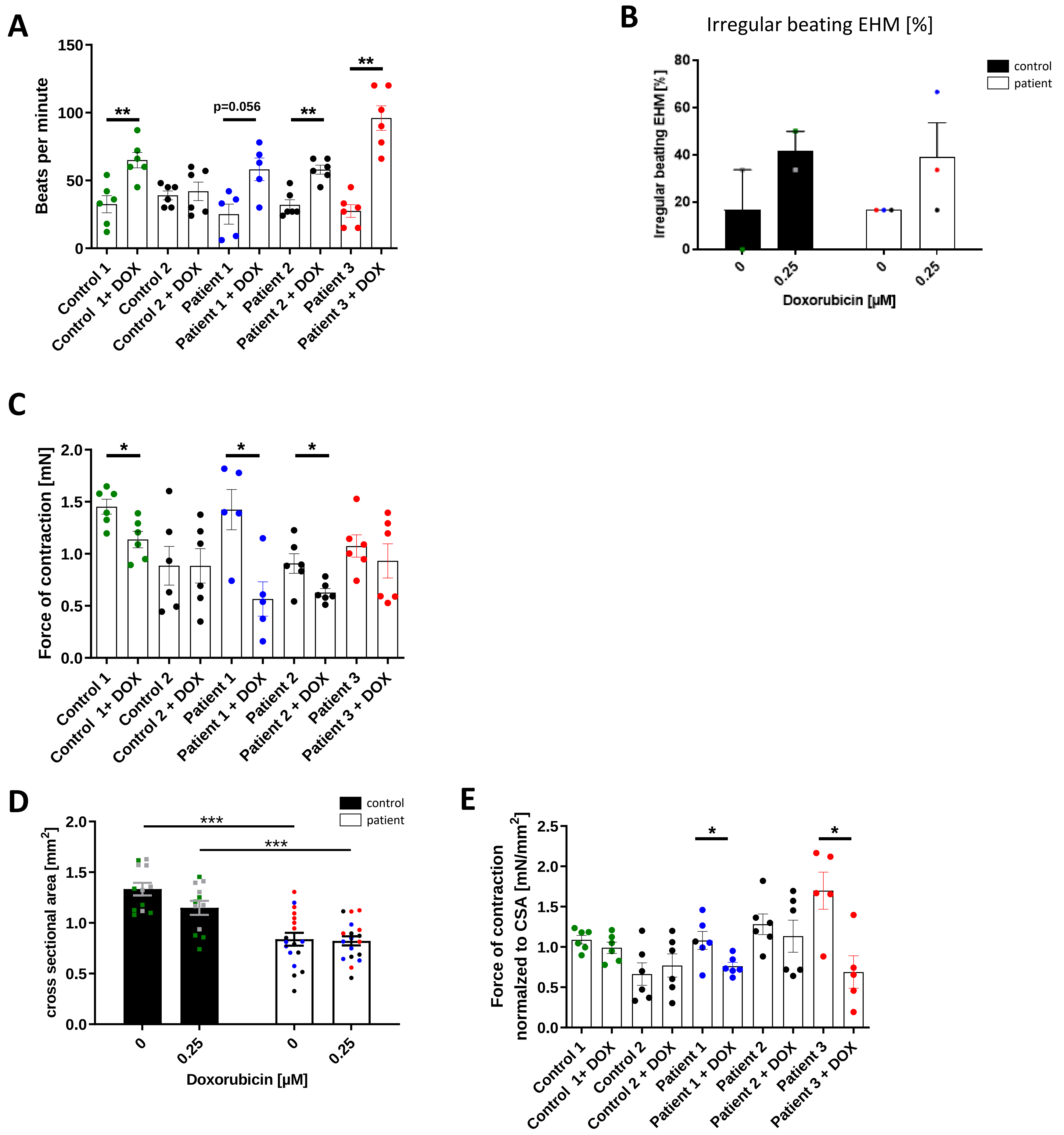

Supplementary Figure 8:

A

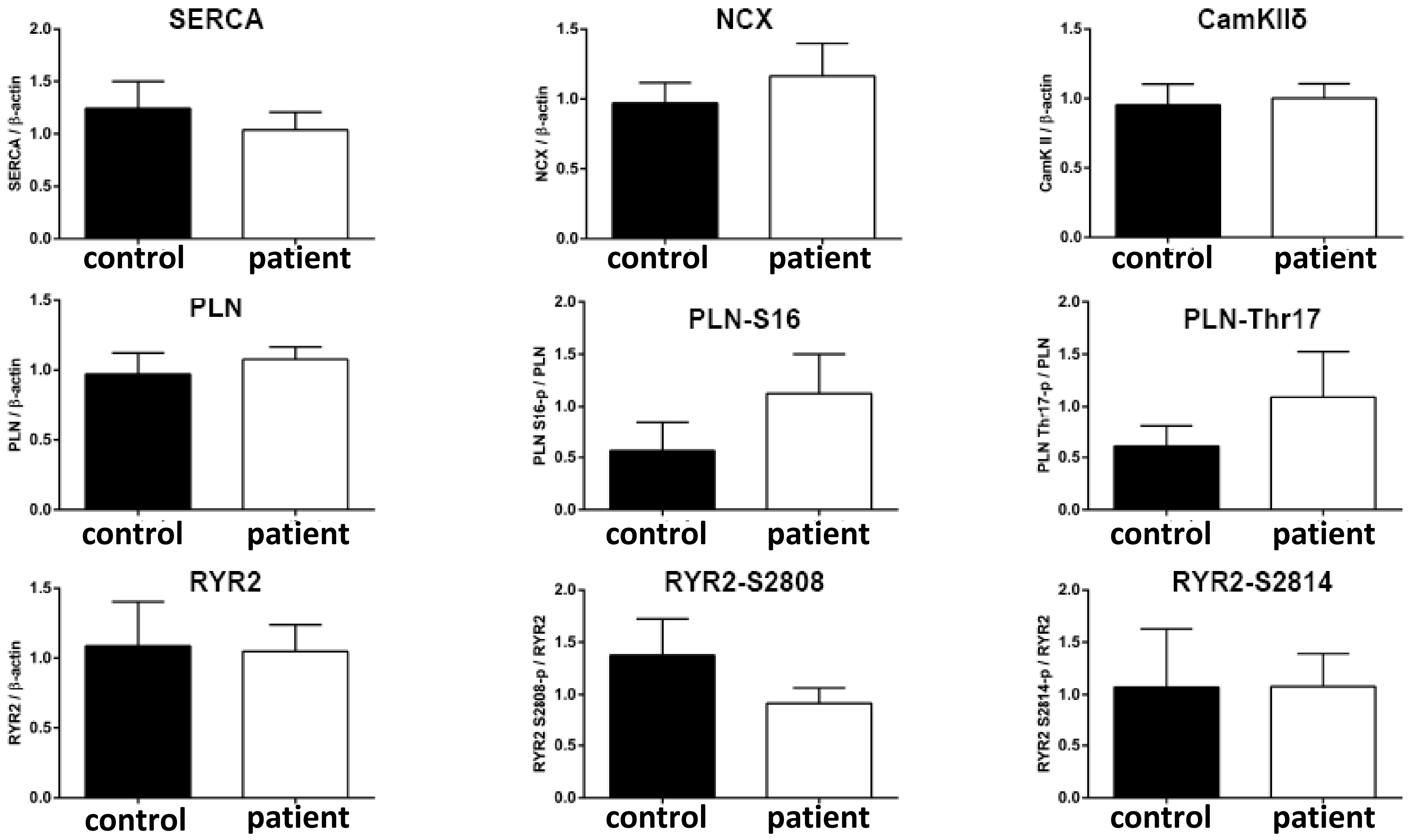

B

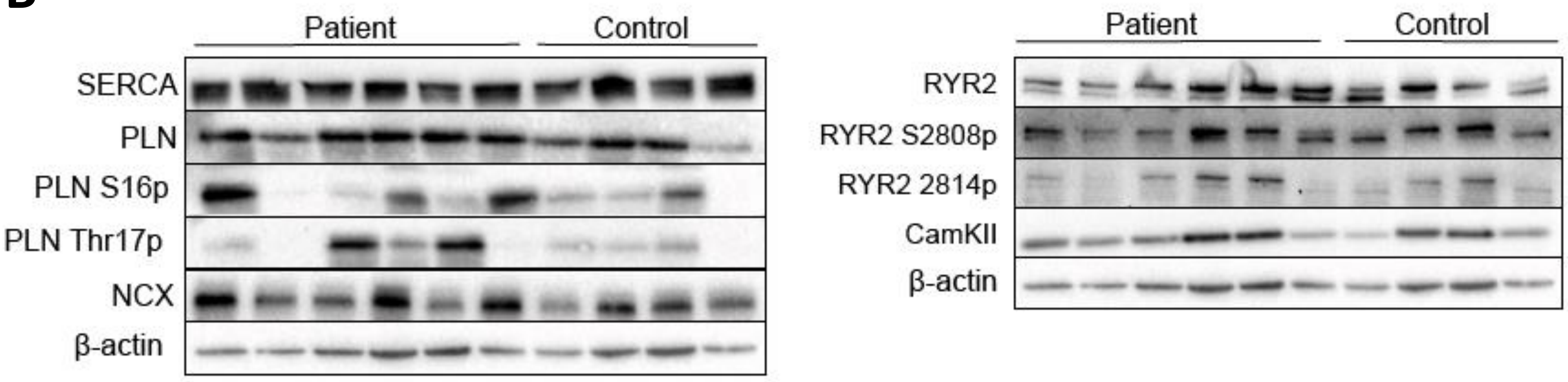

Supplement: Supplementary file 9 — Supplementary file9 (PDF 878 KB) [file 395_2022_918_MOESM9_ESM.pdf]
